# Resilience factors and mechanisms in the coal power supply chain: A quantitative analysis using fuzzy DEMATEL, ISM, and ANP methods

**DOI:** 10.1371/journal.pone.0322952

**Published:** 2025-06-02

**Authors:** Yingchen Wang, Mingkang Li, Linlin Sun, Jiaqing Liu, Yiran Wang

**Affiliations:** School of Management Engineering and Business, Hebei University of Engineering, Handan, Hebei Province, China; University of Salamanca: Universidad de Salamanca, SPAIN

## Abstract

Under the influence of global energy market fluctuations, policy changes, and natural disasters, the coal power supply chain faces significant risks. However, existing research on supply chain resilience management, particularly regarding the identification and quantitative analysis of resilience factors in the coal power supply chain, remains insufficient. Therefore, this study aims to identify the key factors influencing the resilience of the coal power supply chain and analyze the mechanisms of interaction between these factors. This study first conducted a literature review to identify 20 factors classified into three dimensions: adaptive capacity, restorative capacity, and absorptive capacity. Secondly, an innovative approach integrating fuzzy DEMATEL, ISM, and ANP was introduced to systematically identify the key influencing factors of coal power supply chain resilience and analyze their mechanisms. The results reveal that electricity demand forecasting capability, government intervention and coordination capacity, and technological level of the coal industry are the primary causal factors affecting supply chain resilience, playing critical roles in ensuring supply chain stability and adaptability. Additionally, factors such as risk prevention and maintenance level and information transmission efficiency also serve as important supporting elements. This research constructs a cause-degree-centeredness diagram and a hierarchical model for coal power supply chain resilience, providing theoretical support and practical recommendations for optimizing the supply chain structure and enhancing the capacity to cope with uncertainties in the coal power industry.

## 1. Introduction

Amid rising uncertainties in the global energy market, challenges posed by climate change, and shifting policy landscapes, the coal power supply chain is confronting unprecedented risks. This growing complexity underscores the critical importance of supply chain resilience in ensuring energy security and stable electricity provision. For instance, during the lead-up to the 2008 Chinese New Year, Southern China experienced cascading risks across the “coal-electricity-transportation” energy supply chain. Severe snowstorms disrupted power supply from substations and transmission lines, causing power outages that interrupted coal mining and railway transportation, with profound repercussions on the national economy and citizens’ daily lives [1]. Similarly, the widespread outbreak of the COVID-19 pandemic in 2020 hindered normal enterprise operations. Extensive transportation restrictions implemented for pandemic control delayed coal transportation, resulting in losses for power companies. Disruptions in coal production and transportation led to shortages in coal inventories at power plants [2]. In the latter half of 2021, severe power shortages emerged across multiple regions in China, prompting some areas to impose electricity rationing, which adversely affected industrial production and residents’ daily activities [3]. The outbreak of the Russia-Ukraine conflict further exacerbated instability in international energy markets, triggering significant price surges in coal and natural gas. Consequently, Chinese power plants reliant on imported coal faced sharp fluctuations in fuel costs. Meanwhile, disruptions in global logistics compounded the challenges, undermining the stability of imported coal supplies.

China’s coal resources are widely distributed, with over 1,200 cities endowed with coal reserves. Many cities have prospered and attained economic affluence due to coal mining and related industries. Notable examples include Ordos in Inner Mongolia, Shenmu in Shaanxi, and Datong in Shanxi, as well as other resource-dependent cities [4].The coal power supply chain represents a cornerstone of China’s electricity generation system. According to data from the National Bureau of Statistics, in 2024, non-fossil fuel electricity approached 40% of the nation’s total power generation. The thermal power generation reached 1.44 billion kilowatt-hours, with a year-on-year growth of 3.8%, accounting for 43.1% of the total power generation.

Against this backdrop, enhancing supply chain resilience to promote their ability to recover swiftly and maintain continuous operation amid uncertainties and disruptions has become a focal point for both academia and industry. The key to improving the resilience of the coal power supply chain lies in identifying and thoroughly understanding the various factors that influence it and the relationship between them. These factors stem not only from internal management and operations but also from external constraints such as environmental conditions, market dynamics, technological advancements, and policies. By systematically identifying and analyzing these factors, it becomes possible to provide both theoretical foundations and practical guidance for coal power enterprises to optimize their supply chain structures and enhance their ability to respond effectively to challenges.

## 2. Literature review

### 2.1. Coal power supply chain research

**Fig 1:**
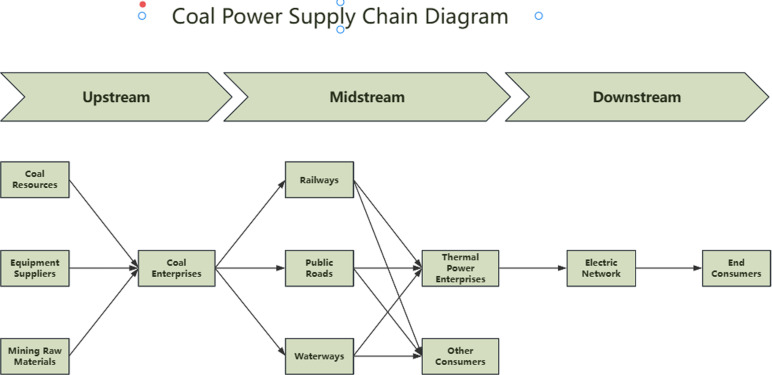
Coal Power Supply Chain Diagram.

Coal power refers to the process of generating electricity using coal as fuel and transmitting it to end-users. The coal power supply chain is a complex system composed of multi-link, multi-entity, multi-stage, and multi-regional nodes [5]. This system can be succinctly described as a sequence: coal production → coal transportation → electricity generation → end-users [1]. These interconnected stages link coal suppliers to ultimate users and are accompanied by significant flows of information and capital [6]. In this process, fuel suppliers deliver coal to power plants, where its thermal energy is converted into electrical energy, which is subsequently transmitted to end-users via the power grid [7]. A specific diagram is shown in Fig 1.

Lin and Bega highlight that while coal power projects under the Belt and Road Initiative have contributed to enhancing power generation efficiency in developing countries, they exhibit notable deficiencies in sustainability [8]. The authors advocate for a gradual transition toward low-carbon energy systems to address these shortcomings. Further research explores the impact of policies and market mechanisms on supply chain dynamics [9]. Their findings emphasize that cash flow and pricing mechanisms are critical to maintaining the stability and operational efficiency of the supply chain.

To achieve carbon reduction targets, researchers have proposed collaborative mechanisms within the supply chain. Wang et al developed a game-theoretic model to analyze supply chain coordination between coal power and renewable energy enterprises under centralized and decentralized decision-making scenarios [10]. Their findings reveal that implementing revenue-sharing and cost-allocation mechanisms can significantly enhance overall supply chain profitability and carbon reduction outcomes. Oberschelp et al. assessed the greenhouse gas and harmful pollutant emissions across the global coal power supply chain [11]. They identified effective mitigation measures, such as improving combustion efficiency, wet flue gas desulfurization, and selective catalytic reduction. Xu et al. examined strategies for coal power plants to enhance clean production, including increasing the preference coefficient for cleaner technologies and reducing the cost coefficient of emission reduction efforts [12]. These strategies were found to effectively improve emission reduction performance and enhance the sustainability of the entire power supply chain. Qiu proposed a green logistics optimization model for the “port-to-plant” coal power supply chain [13]. Grounded in the principles of green logistics, the model employs an improved particle swarm optimization algorithm (IPSO) to achieve efficient, low-emission optimization of coal transportation routes. Sensitivity analyses indicate that the use of efficient and eco-friendly loading and unloading equipment significantly reduces carbon emissions in transportation, providing valuable insights for policymakers and enterprise managers in green supply chain management. Da et al. (2025) provided an important reference for the low-cost decarbonization of coal power in China based on a dynamic optimization approach at the coal power plant level, also offering valuable insights for the global coal power transition [14]. Lou et al. highlighted the significant economic and employment impacts of opening and closing coal power plants, especially the critical role of government policy support during the coal power transformation process [15]. Wang et al. developed dynamic optimization models to explore how flexible technology choices and reasonable policy design can reduce the cost of coal power transformation in China, while providing feasible pathways for coal power transformation in other countries worldwide [16].

### 2.2. Research on supply chain resilience

Recent studies increasingly emphasize the critical importance of supply chain resilience. Supply chain resilience can be defined as the ability of a supply chain to adapt, prepare for unexpected events, respond to disruptions, and restore itself to its expected level of performance and efficiency [17] Several studies have approached the enhancement of supply chain resilience from diverse perspectives, such as focusing on specific risk analyses and their impact on resilience [18] or developing methods to measure resilience [19].

However, only a limited number of studies have outlined general frameworks for constructing resilient supply chains. Notable among these are the frameworks proposed by Yousef [20] and M. Al-Khatib [21], which have demonstrated the applicability of such frameworks across various supply chains. In the context of the coal power supply chain, resilience refers to its ability to recover and maintain normal operations amidst unexpected events, market fluctuations, and environmental changes. In recent years, research on supply chain resilience has gained traction across multiple industries, and the coal power supply chain is emerging as a key area of focus due to its critical role in energy security and economic stability. Enhancing the resilience of the coal power supply chain necessitates addressing multiple uncertainties, including demand fluctuations [22], transportation disruptions [23], fuel supply shortages [24], policy shifts [25], and natural disasters [26]. Particularly in the coal power sector, these uncertainties can have profound economic and social impacts, underscoring the necessity of ensuring the efficient operation and rapid recovery of the supply chain. As such, resilience strategies for the coal power supply chain are of paramount importance for safeguarding energy reliability and socio-economic stability [27].

Jia-guo et al. categorized supply chain resilience capabilities into three dimensions: absorptive capacity, restorative capacity, and adaptive capacity [28]. Restorative capacity refers to the ability of a supply chain to repair and recover following a disruptive event, including the degree and speed of recovery. Absorptive capacity denotes the supply chain’s inherent ability to directly withstand external shocks, encompassing the magnitude and frequency of tolerable disruptions. Adaptive capacity pertains to the supply chain’s ability to adjust and respond to changes in the external environment. Unlike maintaining redundancy, adaptive capacity is characterized by distinct cost and service attributes and does not require the reallocation of redundant resources. In the coal power supply chain, these three dimensions—absorptive, restorative, and adaptive capacities—are closely interconnected. Any disruption at one stage of the chain can cascade through and result in systemic failures across the entire supply chain.

Zhang et al. highlighted that supply-side policy instruments and multi-stakeholder governance mechanisms have gradually emerged as primary strategies for addressing coal power crises [29]. Furthermore, the volatility of market demand has been identified as one of the critical factors influencing supply chain stability. Similarly, Tang emphasized that severe market demand fluctuations exacerbate supply chain pressures, necessitating robust absorptive capacity to maintain stability [30]. In addition, Katsuyuki Nakano believes that climate change affects people’s daily lives and industrial activities by increasing the frequency of extreme weather events such as typhoons and heat waves, rising sea levels, and changing rainfall patterns [31]. These impacts extend to various countries and industries through supply chain disruptions, and supply chains need to have a certain absorption capacity to cope with climate change. Sheffi and Rice demonstrated that the recovery speed of a supply chain following a disruption is a critical indicator of its resilience [32]. In the context of the coal power supply chain, restorative capacity is particularly significant, especially when confronted with natural disasters, policy shifts, or market fluctuations. The enhanced level of service organization development can help enterprises integrate resource supply networks and enhance their ability to move quickly under uncertain and unexpected conditions [33].The availability of backup inventories by fuel suppliers, emergency scheduling in transportation systems, and maintenance strategies of power companies directly influence the efficiency of supply chain recovery. Gu et al. found that information technology can improve the resilience of supply chain [34]. Specifically, in coal transportation, intelligent scheduling systems can expedite recovery processes, ensuring the continuous operation of power companies. Pettit et al. emphasized in their supply chain resilience framework that rapid recovery depends not only on the restoration of physical resources but also on the swift transmission of information and the responsiveness of decision-making systems [35]. Xiaohui proposed that the absorption capacity of coal power supply chain can be improved through digital transformation, so that the supply chain can maintain high elasticity [36].

To enhance supply chain resilience, scholars have proposed various strategies. Christopher and Peck argued that constructing supply chain structures with redundancy and flexibility, strengthening partnerships, and employing dynamic risk management tools are effective methods for improving resilience [37]. In the coal power sector, this entails establishing a more diversified supply network, introducing backup suppliers and alternative transportation routes, and enhancing collaboration with logistics service providers and government agencies to ensure rapid response and recovery during emergencies. Ghobakhloo et.al pointed out that by improving supply chain transparency, flexibility, and collaboration capabilities, resilience functions such as adaptability, responsiveness, and continuity management can be further strengthened [38]. Tan and Pingkuo emphasized the necessity of implementing market-oriented industrial development policies and formulating coordinated industrial development plans [39]. They highlighted the importance of improving the government’s coordination and execution capabilities to manage supply chain risks scientifically while safeguarding China’s energy security. Moreover, they advocated for the effective utilization of fossil and secondary energy resources, alongside the research and promotion of relevant technologies, to facilitate the scaling, industrialization, and integration of the coal power industries.

The current state of domestic and international research indicates that the level of supply chain resilience is shaped by the interplay of multiple factors. While existing studies provide robust frameworks and theoretical foundations for enhancing supply chain resilience, research focusing on the specific factors influencing resilience in coal power supply chains remains relatively limited. Furthermore, although the introduction of multi-criteria decision-making (MCDM) methods has enriched the analytical tools for studying supply chain resilience, challenges such as data acquisition and model complexity hinder their practical application. Given the significant impacts of unexpected events and market fluctuations on coal power supply chains, exploring ways to enhance their resilience is critical for ensuring stable operations. However, existing research has yet to systematically examine the interrelationships among influencing factors in this context. Therefore, this study integrates three methodologies to address this gap: Fuzzy DEMATEL (Decision-Making Trial and Evaluation Laboratory), this method effectively handles uncertainty and ambiguity in expert evaluations. ISM (Interpretive Structural Modeling) constructs hierarchical structures to clarify the dependencies among factors. ANP (Analytic Network Process) is suitable for analyzing complex decision-making scenarios with interdependent factors. By combining these methodologies, the study systematically identifies the key influencing factors of coal power supply chain resilience and analyzes their underlying mechanisms. This integrated approach offers more comprehensive and refined decision-making support for supply chain resilience management.

**Fig 2:**
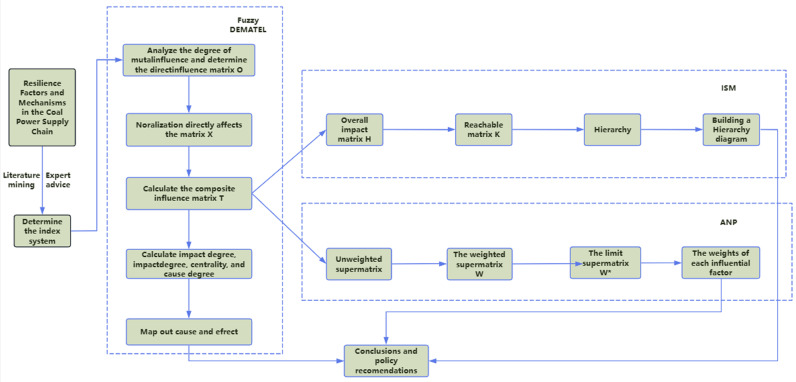
Technical Roadmap.

This study first introduces the core methods and formulas used in Chapter 3, focusing on the use of fuzzy DEMATEL, ISM, and ANP methods to analyze the influencing factors of coal power supply chain resilience and their mechanisms. Chapter 4, based on these three methods, delves into the key influencing factors of the coal power supply chain and their interaction mechanisms, revealed the mechanisms of the role of each factor in supply chain resilience. Chapter 5 provides a detailed discussion of the analysis results, with a focus on interpreting the key influencing factors of coal power supply chain resilience and offer recommendations for improving supply chain resilience in the coal power industry. In Chapter 6, we summarize the main conclusions and implications of the study, while also pointing out the directions for future research. The technical roadmap is shown in Fig 2.

## 3. Research method

This study adopts a combination of fuzzy DEMATEL, ISM, and ANP methods to comprehensively analyze the influencing factors of coal power supply chain resilience and their interactions. The introduction to the Fuzzy DEMATEL-ISM-ANP method are shown in Table 1.

**Table 1:** Introduction to the Fuzzy DEMATEL-ISM-ANP Method.

Method	Key Steps	Main Features	Limitations
Fuzzy DEMATEL	1. Construct the influence factor matrix; 2. Calculate the comprehensive impact degree; 3. Draw causal relationship-centrality diagram.	Suitable for handling causal relationships in complex systems and addressing uncertainties in expert evaluations.	Relies on expert subjective judgment, which may introduce bias and result in incomplete causal relationship analysis.
ISM	1. Construct the influence factor matrix; 2. Calculate the reachability matrix; 3. Build a multi-level hierarchical model.	Suitable for analyzing complex causal relationships and revealing the hierarchy and dependency between factors.	Depends on the accuracy of the influence factor matrix; if factors are not clearly defined or data is incomplete, the results may be affected.
ANP	1. Construct the super matrix; 2. Calculate the weighted super matrix; 3. Perform iterative calculations for final weights.	1. Construct the super matrix; 2. Calculate the weighted super matrix; 3. Perform iterative calculations for final weights.	Expert judgment may introduce bias, and the calculation complexity is high. Large data sets may lead to cumbersome solutions.

The combination of these three methods allows this study to conduct an in-depth analysis of the influencing factors of coal power supply chain resilience from multiple perspectives and levels, ensuring the comprehensiveness and scientific rigor of the research results. Fuzzy DEMATEL helps to reveal causal relationships, ISM provides the hierarchical structure, and ANP further quantifies the importance and interdependence of each factor based on this foundation. Therefore, the use of these three methods effectively overcomes the limitations of a single method and enhances the accuracy and reliability of the research.

### 3.1. Fuzzy DEMATEL method introduction

The most prominent feature of DEMATEL (Decision-Making Trial and Evaluation Laboratory) lies in its ability to perform quantitative analysis of causal relationships. Wu and Lee highlighted that DEMATEL can generate cause-and-effect diagrams based on the prominence-centrality relationship [40]. These diagrams provide decision-makers with intuitive causal chain insights, enabling them to identify critical factors within complex problems. DEMATEL has been widely applied in various fields, including supply chain management, risk management, and technology selection.

As understanding of uncertainty and ambiguity in complex systems deepens, fuzzy set theory has been integrated into the DEMATEL method to enhance its decision-making capabilities in uncertain environments. The traditional DEMATEL approach assumes that decision-makers can precisely evaluate the mutual influence between factors. However, in reality, many decision-making processes involve inherent uncertainty and ambiguity. Consequently, the application of the fuzzy DEMATEL method enables a more accurate representation of this uncertainty, thereby improving the reliability and robustness of the analysis.

Lin and Wu integrated fuzzy set theory with the DEMATEL method and proposed the fuzzy DEMATEL approach [41]. By employing triangular fuzzy numbers to represent the uncertainty and ambiguity in expert opinions, the fuzzy DEMATEL method provides greater flexibility in addressing complex decision-making problems. This approach has been successfully applied to areas such as supply chain risk assessment and product development. Chang et al. explored the application of fuzzy DEMATEL in supply chain management [42]. Their findings demonstrated that the method effectively uncovers the interactions among risk factors and provides decision-makers with more precise causal relationship analyses.

The Steps of Fuzzy DEMATEL Are as Follows:

Step 1: Construct Influencing Factor Indicators and Expert Semantic Conversion Table, as shown in Table 2.

**Table 2:** Influencing Factors and Expert Semantic Conversion Table.

Semantic Variable	Expert Score	Triangular Fuzzy Number (TFN)
No Influence (N)	0	(0.00,0.00,0.25)
Low Influence (L)	1	(0.00, 0.25, 0.50)
Moderate Influence (M)	2	(0.25, 0.50, 0.75)
High Influence (H)	3	(0.50, 0.75, 1.00)
Very High Influence (VH)	4	(0.75, 1.00, 1.00)

Step 2: Construct the Direct-Relation Matrix

First, based on expert knowledge and experience, the relationships between two influencing factors are determined. The relationship between factors can be categorized into five levels. In fuzzy DEMATEL, experts use triangular fuzzy numbers $\;{\tilde{a}}_{ij}=\left(a_{ij}^{L},a_{ij}^{M},a_{ij}^{U}\right)$ to represent the uncertain relationship between two factors, where: $a_{ij}^{L}$ is the lower bound, representing the minimum influence. $a_{ij}^{M}$ is the most likely value, representing the average or expected influence. $a_{ij}^{U}$ is the upper bound, representing the maximum possible influence.

The triangular fuzzy number is used to represent the influence relationship among various factors, and then the direct influence matrix Z obtained by defuzzification method is:


$$Z=\begin{array}{ccc} {}*20cz_{11} & \ldots & z_{1j}\\ \ldots & \ldots & \ldots \\ z_{i1} & \ldots & z_{ij} \end{array}$$

Step 3: Based on the normalization formula, the direct influence matrix is normalized to obtain the canonical influence matrix X ($X={\left(x_{ij}\right)}_{n\times n}$).


$$X=\frac{1}{\underset{1\leq i\leq n}{\max}\sum {\mathrm{\backslash nolimits}}_{j=1}^{n}f_{ij}}F={\left(x_{ij}\right)}_{n\times n},i,j=1,2,\cdots ,n$$ (1)

It is known that $0\leq x_{ij}\leq 1$, and $\underset{1\leq i\leq n}{\max}\sum_{j=1}^{n}x_{ij}=1$.

Step 4: Calculate the comprehensive influence matrix T($T={\left(t_{ij}\right)}_{n\times n}$), the calculation formula is as follows: W


$$T=(X+X_{2}+\ldots +X_{k})=X{\left(I-X\right)}^{-1}$$ (2)

Where I is the identity matrix.

Step 5: Calculate the impact and impact of each factor.

By summing the elements of the comprehensive influence matrix row by row, the influence degree of the corresponding factors is obtained; by summing the elements of the comprehensive influence matrix column by column, the affected degree of the corresponding factors is obtained.


$$D=\sum_{j=1}^{n}t_{ij},i=1,2,\ldots ,n$$ (3)

$$C=\sum_{i=1}^{n}t_{ij},j=1,2,\ldots ,n$$ (4)

Step 6: Calculate the centrality and causality of each factor.

Centrality is obtained by adding the influence degree and the affected degree, while the degree is obtained by subtracting the affected degree from the influence degree.


M=D+C (5)

R=D−C

Step 7: Plotting the degree-of-causality-centrality graph

The horizontal axis of a Degree-of-Causality-Centrality diagram is D + C and the vertical axis is D-C. The causality-centrality diagram can simplify the complex cause and effect relationship, which can help to analyze and solve the problem more intuitively and deeply. In addition, through this diagram, decision makers can clearly identify which factors belong to the “cause category” (i.e., factors that trigger changes in other factors) and which belong to the “effect category” (i.e., factors that are influenced by other factors). Based on these analyses, decision makers are able to make decisions and optimize them according to the characteristics of different types of factors.

### 3.2. ISM method introduction

Interpretive Structural Modeling (ISM), initially proposed by Professor Warfield in 1973, enables the conversion of ambiguous and uncertain concepts into an intuitive and tangible framework characterized by comprehensive structural relationships. ISM is adept at constructing structural models, thereby serving as a foundational tool for system analysis. It is particularly well-suited for investigating and analyzing systems that encompass a multitude of variables, exhibit complex and indistinct causal relationships, and possess unclear structural configurations. Consequently, ISM provides a robust basis for subsequent program classification and the optimization of decision-making processes.

Shin and Par employed ISM in conjunction with a systematic literature review to identify and design key indicators of supply chain resilience, analyzing their interdependencies and hierarchical relationships [43]. Their study categorized critical resilience factors and developed a model comprising four types of influence variables: driver, dependent, autonomous, and linkage factors. Attia and Uddin applied ISM as part of a hybrid assessment methodology to identify and analyze critical resilience factors in supply chains, with a particular focus on risk management and inter-organizational collaboration. ISM facilitated the analysis of the energy supply chain’s adaptability and response strategies under uncontrollable external factors, providing a framework to enhance the resilience of energy systems [44].

The steps for ISM calculation are as follows:

Step 1: Calculate the overall impact matrix H($H={\left(h_{ij}\right)}_{n\times n}$)


H=T+I (6)

Where matrix I is the unit matrix.

Step 2: Calculate the reachable matrix K.

To determine the threshold value λ, it is calculated based on a statistical distribution method as: λ=μ+ν. Here, μ and ν represent the mean and standard deviation of all elements in matrix *T*, respectively. μ and ν are calculated as:


$$\mu =\frac{\sum {\mathrm{\backslash nolimits}}_{i=1}^{n}\sum {\mathrm{\backslash nolimits}}_{j=1}^{n}t_{ij}}{n^{2}}$$ (7)

$$v=\sqrt{\frac{\sum {\mathrm{\backslash nolimits}}_{i=1}^{n}\sum {\mathrm{\backslash nolimits}}_{j=1}^{n}{\left(t_{ij}-\mu \right)}^{2}}{n^{2}}}$$ (8)

Based on λ the reachability matrix K is obtained from the integrated impact matrix T. The reachability matrix K is calculated using the formula:


$$\begin{array}{c} k_{ij}=\{\begin{array}{c} @\;\mathrm{-2pt}l1\;\;\\ 0\;\; \end{array}\begin{array}{c} {}*20ct_{ij}>\lambda \\ t_{ij}\leq \lambda \end{array} \end{array}$$ (9)

Step 3: Determine the reachable set Q, the prior set A, and the intersection set to construct a multilevel recursive order structure model of the influencing factors.


$$Q\left(P_{i}\right)=\left\{P_{i}|P_{i}\in P,k_{ij}=1\right\}$$ (10)

$$A\left(P_{j}\right)=\left\{P_{j}|P_{j}\in P,k_{ji}=1\right\}$$ (11)

### 3.3. ANP method introduction

The Analytic Network Process (ANP), developed by Saaty in 1996 as an extension of the Analytic Hierarchy Process (AHP), is designed to address decision-making problems characterized by complex interdependencies. Unlike AHP, which assumes a hierarchical structure for decision problems, ANP allows for feedback and mutual influence among decision elements. This flexibility makes ANP particularly effective for tackling complex real-world decision problems. ANP analyzes the interdependence among decision elements by constructing a supermatrix, which captures the relationships and interactions among these elements. It then uses weights derived from the supermatrix to prioritize multiple alternatives in the decision-making process. By accommodating multi-dimensional and interrelated decision problems, ANP has demonstrated exceptional performance in various application areas, including supply chain management, risk analysis, and project management.

The D-ANP model [45], integrates DEMATEL and ANP to enhance decision-making processes. DEMATEL, an effective method for analyzing causal relationships [46], provides a quantitative representation of criteria and considers structural models, ultimately generating a comprehensive influence matrix [47]. In the D-ANP approach, the comprehensive influence matrix produced by DEMATEL is directly utilized as the unweighted supermatrix for ANP, streamlining the process by avoiding the tedious pairwise comparisons typically required in ANP [48]. This integration allows D-ANP to leverage the strengths of both methods, improving efficiency and accuracy in multi-criteria decision-making problems.

The comprehensive influence matrix T is regarded as the unweighted supermatrix [49]. Subsequently, it is normalized to obtain the weighted supermatrix W. The weighted supermatrix W is then subjected to multiple iterations of self-multiplication until convergence is achieved, resulting in the final W supermatrix, which represents the global weights of all elements. When identifying critical factors, it is essential to consider the importance rankings derived from both the total influence matrix T and the final supermatrix W [50]. This iterative process ensures that interdependencies and feedback among the elements are accurately reflected, enabling a robust prioritization of key factors.

The steps of ANP are as follows:

Step 1: Compute the weighted supermatrix


$$W_{ij}=\frac{T_{ij}}{\sum {\mathrm{\backslash nolimits}}_{i=1}^{n}T_{ij}}$$ (12)

Step 2: Calculate the limit supermatrix


$$\underset{k\to \infty }{\lim}W^{k}=W^{*}$$ (13)

## 4. Coal power supply chain resilience influencing factors and mechanisms

### 4.1. Data sources

This study identifies 20 factors influencing the resilience of the coal power supply chain through a combination of literature review. These factors are primarily categorized into three dimensions: adaptive capacity, recovery capacity, and absorptive capacity. The coal power supply chain, as a vast and intricate system, exhibits complex interactions among these resilience factors. Understanding these interdependencies is essential for analyzing the mechanisms that contribute to the system’s overall resilience and for developing effective strategies to enhance its robustness and sustainability.

The specific influencing factors and their quantities are detailed in Table 3.

**Table 3:** Influencing Factors.

Primary Indicators	Secondary Indicators	Description	
Restorative Capacity (Ability to recover after sudden disruptions)	Risk Prevention and Maintenance Level (A1)	Preventive measures taken by enterprises and the government to address supply chain disruption risks, including equipment maintenance, transportation management, and disaster prevention.	[51]
	Electricity Demand Forecasting Capability (A2)	The ability of power companies to accurately forecast demand based on historical data and market changes to ensure a balance between production and consumption.	[22]
	Grid Dispatching Capability (A3)	The ability of the power dispatch center to allocate power resources during demand fluctuations to ensure stable power supply.	[52]
	Coal Emergency Reserve Assurance Capability (A4)	The capability to efficiently utilize emergency coal reserves during critical moments, including logistics and reserve facilities.	[53]
	Electricity Storage Technology Level (A5)	Efficient electricity storage technologies that help balance grid supply and demand during demand fluctuations.	[54]
	Electricity Emergency Dispatch System (A6)	The emergency dispatch system of the grid in response to sudden incidents, including the coordinated dispatch of various power production units.	[55]
	Government Intervention and Coordination Capability (A7)	The ability of the government to coordinate and allocate the market in emergencies, ensuring stable supply chain operations.	
Absorptive Capacity (Ability to directly withstand external shocks)	Risk Perception Capability (B1)	The ability of enterprises to perceive and predict market fluctuations, energy price changes, and potential supply disruptions.	[56]
	Non-State Economic Development Level (B2)	The extent and depth of participation of non-state economies in the coal power supply chain, increasing market diversity and resilience.	[22]
	Diversity of Enterprise Emergency Actions (B3)	The variety of emergency measures taken by enterprises during supply chain disruptions.	[57]
	Relationship between Enterprises and Service Organizations (B4)	The impact of cooperation between enterprises and logistics, technical services, and other key service organizations on supply chain resilience.	[58]
	Information Transmission Efficiency (B5)	The speed and accuracy of information transmission between nodes in the supply chain.	[59]
	Asset Liquidity (B6)	The liquidity and flexibility of enterprise assets to quickly allocate resources to respond to emergencies in the face of risks or fluctuations.	[60]
	Relevant Legal and Policy Level (B7)	The impact of changes in government policies and regulations related to the coal and power industries on supply chain management.	[25]
Adaptive Capacity (Ability to adjust and adapt to changes in the external environment)	Degree of Government Intervention in the Market (C1)	The extent of government regulation in the coal power supply chain, such as market regulation and price intervention measures.	[61]
	Technological Level of the Coal Industry (C2)	The support provided by advanced coal production technology to supply chain resilience.	[62]
	Development Level of Service Organizations (C3)	The level of support and assurance provided by third-party services related to the supply chain.	[63]
	Industry Resource Competitiveness (C4)	The competitive advantage of the coal power industry in regional markets.	[64]
	Technological Innovation Capability (C5)	The capability of enterprises to innovate technology within the coal power industry.	[65]
	Product Market Development Level (C6)	The maturity and development level of coal and related energy product markets.	[66]

### 4.2. Fuzzy DEMATEL analysis results

The questionnaire was distributed to 24 experts from coal and electricity companies, all of whom have at least 5 years of experience in the coal power industry or are academic experts in the field. These experts were invited to assess the interactions of the influencing factors of coal power supply chain resilience. A total of 20 valid responses were received, yielding a response rate of 83.33%.The specific information can be found in Table 4.

**Table 4:** Demographics.

Measures	Groups	Frequency	Percentage (%)
Gender	Male	12	60
	Female	8	40
Age	30-40	5	25
	41-50	11	55
	51or more	4	20
Education	Bachelor’s degree	14	70
	Postgraduate	6	30
Experience	5-10 years	7	35
	10-15 years	9	45
	15 years or more	4	20

**Fig 3:**
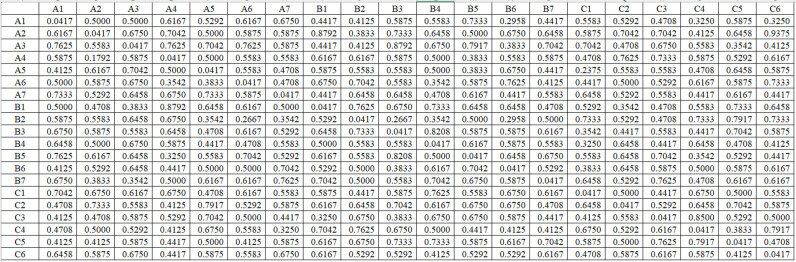
Direct Influence Matrix O.

The triangular fuzzy number was used to represent the influence relationship between the factors and then the direct influence matrix obtained by defuzzification is shown in Fig 3.

Then, by normalizing the matrix using Formula 1, the standardized direct influence matrix is obtained.

Then the integrated impact matrix T is calculated based on the normalized direct impact matrix Z. The integrated impact matrix T is obtained by MATLAB software.

Summing the factors in the integrated influence matrix T by rows, which indicates the combined influence value of the corresponding factor in each row on the other factors. Summing the factors of the integrated influence matrix by columns yields the influenced degree, which represents the combined influence value of the corresponding factor in each column on the other factors. Add the influence degree and the influenced degree to get the center degree, and subtract to get the cause degree, The results are shown in Table 5.

**Table 5:** Comprehensive impact table of factors affecting coal power supply chain resilience.

Factor	Influence degree	Influenced degree	Centrality degree	Centrality ranking	Cause degree
A1	7.3186	8.2223	15.5410	19	-0.9037
A2	9.0547	7.5083	16.5630	5	1.5465
A3	8.6479	8.4053	17.0532	1	0.2425
A4	7.8572	7.9303	15.7875	15	-0.0732
A5	7.5402	7.9907	15.5309	20	-0.4505
A6	7.8322	7.9688	15.8011	14	-0.1366
A7	8.1893	7.8353	16.0245	11	0.3540
B1	8.3448	8.2581	16.6029	4	0.0867
B2	7.4488	8.2994	15.7482	17	-0.8506
B3	8.3231	8.5300	16.8531	2	-0.2069
B4	7.6581	8.3244	15.9824	12	-0.6663
B5	8.3783	8.3795	16.7578	3	-0.0012
B6	7.7583	7.9514	15.7097	18	-0.1932
B7	8.3994	7.8054	16.2048	8	0.5940
C1	8.3873	7.4798	15.8672	13	0.9075
C2	8.6703	7.8753	16.5457	6	0.7950
C3	7.6673	8.3624	16.0297	10	-0.6951
C4	7.7661	7.9972	15.7633	16	-0.2311
C5	8.3143	8.1760	16.4903	7	0.1382
C6	7.9562	8.2123	16.1684	9	-0.2561

**Fig 4:**
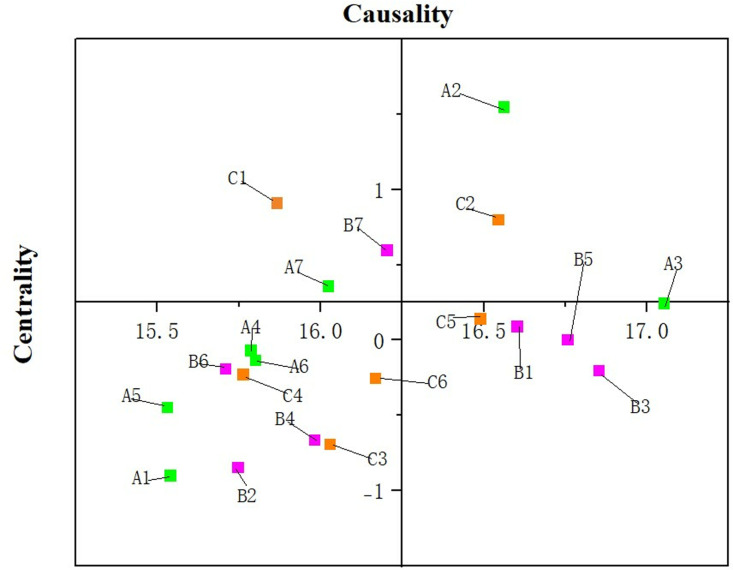
Coal power supply chain elasticity cause-degree-centeredness diagram.

Plotting the degree of cause-centeredness of coal power supply chain resilience, see Fig 4.

In the coal power supply chain resilience system, the results of the fuzzy DEMATEL analysis demonstrate the different relationships of influence from the perspectives of centrality and causality. From the centrality perspective, grid dispatching capability (A3), diversity of corporate emergency responses (B3), and efficiency of information transmission (B5) emerge as pivotal factors. These elements frequently interact with others, serving as critical nodes for coordinated operations.

From the perspective of causality, electricity demand forecasting capability (A2), the degree of government intervention in the market (C1), and technological level of the coal industry (C2) are identified as the primary causal factors. As a core driving force in the coal power supply chain resilience system, electricity demand forecasting capability (A2) facilitates proactive preparation for future demand fluctuations and enhances the system’s capacity for rational resource allocation. Meanwhile, government intervention in the market (C1) provides essential policy support during market volatility, ensuring the stable operation of the supply chain. Furthermore, advancements in the technological level of the coal industry (C2) strengthen the supply chain’s adaptability to market conditions and boost production efficiency, laying a robust technological foundation for sustainable development. Moreover, the DEMATEL analysis results reveal the roles of several outcome-oriented factors, such as risk prevention and maintenance level (A1), asset liquidity (B6), and electricity storage technology level (A5). These factors exhibit lower causality, functioning primarily as responses to other factors.

In summary, the key to system optimization lies in prioritizing the enhancement of electricity demand forecasting capability (A2), government intervention capability (C1), and technological level of the coal industry (C2) to drive the overall improvement of coal power supply chain resilience. At the same time, it is essential to ensure the efficient operation of centrality factors such as grid dispatching capability (A3), diversity of corporate emergency actions (B3), and information transmission efficiency (B5) to safeguard the supply chain’s coordination and emergency response capabilities. Additionally, outcome-oriented factors such as risk prevention and maintenance level (A1) should undergo continuous optimization to bolster the resilience of the coal power supply chain in uncertain environments. By balancing the interplay between causal factors and outcome-oriented factors, the coal power supply chain can achieve a higher level of coordinated operation and sustainable development, preparing effectively for future market challenges.

### 4.3. Analysis of ISM results

Calculated according to Formula 9, the reachability matrix K is obtained and is shown in Table 6.

**Table 6:** Coal Power Supply Chain Resilience Influencing Factors Reachable Matrix K.

	A1	A2	A3	A4	A5	…...	C2	C3	C4	C5	C6
A1	1	0	0	0	0		0	0	0	0	0
A2	1	1	1	1	1		1	1	1	1	1
A3	1	0	1	1	1		0	1	0	0	0
A4	0	0	0	1	0		0	0	0	0	0
A5	0	0	0	0	1		0	0	0	0	0
…...						…...					…...
C2	1	0	1	1	1		1	1	1	1	1
C3	0	0	0	0	0		0	1	0	0	0
C4	0	0	0	0	0		0	0	1	0	0
C5	0	0	0	0	0		0	1	0	1	0
C6	0	0	0	0	0		0	0	0	0	1

The hierarchical division in the Interpretive Structural Modeling (ISM) process involves identifying the reachable set, antecedent set, and intersection set. In the reachability matrix, all factors corresponding to rows with a value of 1 constitute the reachable set, denoted as Q. Similarly, all factors corresponding to columns with a value of 1 in the reachability matrix form the antecedent set, denoted as A. The intersection set is the common elements shared between the reachable set and the antecedent set, expressed asQ∩A. This relationship is formalized in Formula 10 and Formula 11, the results are shown in Table 7.

**Table 7:** Reachable Set, Antecedent Set, and Intersection Set for the First Level.

Indicators	Reachable set	Precedence set	Intersection
A1	[A1]	[A1, A2, A3, B1, B5, B7, C1, C2]	[A1]
A2	[A1, A2, A3, A4, A5, A6, A7, B1, B2, B3, B4, B5, B6, B7, C2, C3, C4, C5, C6]	[A2]	[A2]
A3	[A1, A3, A4, A5, A6, B2, B3, B4, B5, C3]	[A2, A3, B1, B5, B7, C1, C2]	[A3, B5]
A4	[A4]	[A2, A3, A4, B1, B5, B7, C1, C2]	[A4]
A5	[A5]	[A2, A3, A5, B1, B5, B7, C1, C2]	[A5]
A6	[A6]	[A2, A3, A6, B1, B5, B7, C1, C2]	[A6]
A7	[A7, B2, B3, B4]	[A2, A7]	[A7]
B1	[A1, A3, A4, A5, A6, B1, B2, B3, B4, B5, C3, C5]	[A2, B1, B7, C2]	[B1]
B2	[B2]	[A2, A3, A7, B1, B3, B5, B7, C1, C2, C5]	[B2]
B3	[B2, B3, B4]	[A2, A3, A7, B1, B3, B5, B7, C1, C2, C5, C6]	[B3]
B4	[B4]	[A2, A3, A7, B1, B3, B4, B5, B7, C1, C2, C5]	[B4]
B5	[A1, A3, A4, A5, A6, B2, B3, B4, B5, C3]	[A2, A3, B1, B5, B7, C1, C2]	[A3, B5]
B6	[B6]	[A2, B6, C2]	[B6]
B7	[A1, A3, A4, A5, A6, B1, B2, B3, B4, B5, B7, C3, C5]	[A2, B7]	[B7]
C1	[A1, A3, A4, A5, A6, B2, B3, B4, B5, C1, C3]	[C1]	[C1]
C2	[A1, A3, A4, A5, A6, B1, B2, B3, B4, B5, B6, C2, C3, C4, C5, C6]	[A2, C2]	[C2]
C3	[C3]	[A2, A3, B1, B5, B7, C1, C2, C3, C5]	[C3]
C4	[C4]	[A2, C2, C4]	[C4]
C5	[B2, B3, B4, C3, C5]	[A2, B1, B7, C2, C5]	[C5]
C6	[C6]	[A2, C2, C6]	[C6]

From the above table, it can be determined that the first-level influencing factors include A1, A4, A5, A6, B2, B4, B6, C3, C4, and C6. The corresponding rows and columns for these factors should be removed from the table. According to the Formula 10 and Formula 11, step by step, and finally get six layers. The results are shown in Table 8.

**Table 8:** Reachable Set, Antecedent Set, and Intersection Set for the Sixth Level.

Indicators	Reachable set	Precedence set	Intersection
A2	[A2]	[A2]	[A2]

**Fig 5:**
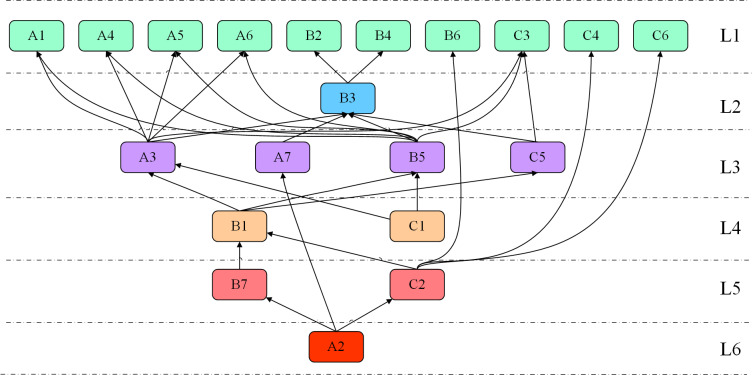
Hierarchical Structure Diagram of Influencing Factors in Coal Power Supply Chain Resilience.

The sixth-level influencing factor is A2, marking the conclusion of the UP hierarchical structuring process. The hierarchical levels are as follows: First Level (L1): {A1, A4, A5, A6, B2, B4, B6, C3, C4, C6}; Second Level (L2): {B3}; Third Level (L3): {A3, A7, B5, C5}; Fourth Level (L4): {B1, C1}; Fifth Level (L5): {B7, C2}; Sixth Level (L6): {A2}. The hierarchical structure diagram is shown in Fig 5.

In the coal power supply chain resilience system, various factors interact across different levels to collectively influence the supply chain’s resilience and its ability to respond to risks. Based on the ISM model analysis, these factors are categorized into six levels, which are closely interconnected, forming a complex system structure. L1 represents the direct layer, L2-L5 are intermediate layers, and L6 constitutes the fundamental layer.

In the first layer (L1), factors include risk prevention and maintenance level, coal emergency reserve assurance capability, electricity storage technology level, electricity emergency dispatch system, non-state economic development level, relationship between enterprises and service organizations, asset liquidity, development level of service organizations, industry resource competitiveness, and product market development level. These direct factors play a critical role in the operation of the supply chain, primarily manifesting in areas such as energy reserves, system dispatching, market competitiveness, and enterprise resource relationships. Robust technological capabilities and market competitiveness ensure the efficient operation of the supply chain. Additionally, the development of the non-state economy and the liquidity of assets enhance the system’s flexibility and resilience, enabling it to better adapt to external changes.

At the second layer, the diversity of corporate emergency actions serves as a critical bridge, reflecting a firm’s ability to implement various emergency strategies in response to unexpected events. By adopting flexible emergency plans, companies can swiftly adjust their operational strategies, thereby enhancing the adaptability and resilience of the supply chain to maintain stable operations under challenging conditions. The third layer encompasses grid dispatching capability, government intervention and coordination, information transmission efficiency, and technological innovation capability. These elements significantly influence the overall operational efficiency and responsiveness of the system. Effective grid dispatching ensures the rational allocation of energy resources; government intervention and coordination provide essential support for market stability and supply chain reliability; and both efficient information transmission and continuous technological innovation contribute to reducing response times and establishing a robust foundation for long-term system development.

In the fourth layer, risk perception capability and the degree of government intervention in the market reflect the system’s ability to provide early warnings and implement market regulation before risks materialize. Risk perception capability enables the supply chain to identify potential threats, while government intervention plays a critical regulatory role in addressing market failures, ensuring the rational allocation of resources and the stable operation of the supply chain. In the fifth layer, relevant legal and policy level and technological level of the coal industry serve as vital support factors for the supply chain. Comprehensive legal and policy frameworks provide institutional safeguards for enterprise operations, while advancements in the coal industry’s technological level enhance production efficiency and market competitiveness, laying a solid foundation for the system’s robust performance.

In the sixth layer, electricity demand forecasting capability stands as the fundamental factor, exerting a profound impact on the overall planning and operation of the supply chain. Accurate electricity demand forecasting enables enterprises to formulate production and dispatching plans in advance, thereby preventing resource wastage or supply shortages caused by demand fluctuations. This capability serves as the cornerstone of supply chain stability, ensuring efficient operations under complex and dynamic conditions.

In summary, these factors significantly influence the resilience of the coal power supply chain across multiple dimensions, including technology, market, management, policy, and environment. Enhancing supply chain resilience requires coordinated optimization at every level. At the first level, enterprises should improve coal reserve management and electricity storage technologies to ensure adequate resources and emergency systems [67–70]. At the intermediate layers, both governments and enterprises must jointly enhance information transmission efficiency and technological innovation capability [71] to foster flexible market development. At the fundamental level, improving electricity demand forecasting capability will provide a solid foundation for the stable operation of the supply chain. By coordinating the relationship between causal factors and result-oriented factors, the coal power supply chain will be able to achieve a higher level of coordinated operation and sustainable development, thereby being well-prepared to address market challenges.

### 4.4. Analysis of ANP results

The integrated influence matrix generated by DEMATEL is directly used as the unweighted supermatrix for ANP. The weighted supermatrix is then calculated using Formula 12.

The resulting weighted supermatrix is then used to calculate the limiting supermatrix by using Formula 13.

According to the limit supermatrix obtained, the weights of each influential factor of coal power supply chain elasticity can be obtained, as shown in Table 9.

**Table 9:** Weights of factors influencing the resilience of the coal power supply chain.

Primary Indicators	Primary Indicators weight	Secondary Indicators	Secondary Indicators weight	Weight sorting
Restorative Capacity	0.3494	A1	0.0454	20
		A2	0.0560	1
		A3	0.0535	3
		A4	0.0486	12
		A5	0.0467	18
		A6	0.0485	13
		A7	0.0507	10
Absorptive Capacity	0.3487	B1	0.0516	7
		B2	0.0462	19
		B3	0.0515	8
		B4	0.0474	17
		B5	0.0519	6
		B6	0.0481	15
		B7	0.0520	4
Adaptive Capacity	0.3019	C1	0.0519	5
		C2	0.0537	2
		C3	0.0475	16
		C4	0.0481	14
		C5	0.0515	9
		C6	0.0493	11

Based on the results of the ANP analysis, the weight of Restorative Capacity in the resilience system of the coal power supply chain is the highest, followed by Absorptive Capacity, with Adaptive Capacity having the lowest weight.

In terms of Restorative Capacity, the weight of Electricity Demand Forecasting Capability (A2) is the highest, at 0.0560, indicating that accurate demand forecasting is crucial for the efficient recovery of the coal power supply chain’s resilience. By accurately predicting future electricity demand, enterprises can proactively develop production and dispatch plans, preventing resource waste or shortages in the supply chain. Additionally, the weight of Grid Dispatching Capability (A3) is also relatively high (0.0535), suggesting that the coordinated interplay between demand forecasting and resource dispatch is a key factor in ensuring the stable operation of the supply chain during the recovery process. Electricity Storage Technology Level (A5) and Government Intervention and Coordination Capability (A7) also hold significant positions within Restorative Capacity, underscoring the critical roles that technological support and government coordination play in energy security and system restoration.

In terms of Adaptive Capacity, the weight of Technological Level of the Coal Industry (C2) is 0.0537, indicating that technological upgrades have a significant impact on the system’s adaptability. Additionally, the Degree of Government Intervention in the Market (C1) and Technological Innovation Capability (C5) also hold relatively high weights, highlighting the importance of policy support and innovation capabilities in responding to market fluctuations and changes. The enhancement of Adaptive Capacity relies not only on technology and policy but also on the system’s ability to maintain flexibility in the face of market shifts, ensuring the stability and sustainable development of the supply chain.

In terms of Absorptive Capacity, the weights of Information Transmission Efficiency (B5) and Relevant Legal and Policy Level (B7) are 0.0519 and 0.0520, respectively, indicating that an efficient information transmission system and a well-established legal and policy framework enhance the system’s ability to withstand external shocks. The Diversity of Enterprise Emergency Actions (B3) and Risk Perception Capability (B1) also play a proactive role in emergency response, ensuring the system’s flexibility and rapid adjustment during unforeseen events, thus safeguarding the ability to respond swiftly and effectively.

Overall, in the process of enhancing the resilience of the coal power supply chain, priority should be given to strengthening Restorative Capacity, particularly Electricity Demand Forecasting Capability (A2) and Grid Dispatching Capability (A3), to ensure that the coal power supply chain can swiftly recover following disruptions. At the same time, efforts should focus on improving Adaptive Capacity by leveraging advancements in Technological Level of the Coal Industry (C2), Technological Innovation Capability (C5), and Government Intervention in the Market (C1) to enhance the dynamic adaptability of the coal power supply chain’s resilience system. As for Absorptive Capacity, further improvements should be made to the information system and legal policies (B5, B7), while enhancing the collaboration between enterprises and service organizations (B4) to ensure that emergency resources can respond promptly. Moreover, the government should strengthen coordination and promote the sustainable development of both the market and technology, ensuring that the coal power supply chain operates efficiently, stably, and sustainably despite market fluctuations and external shocks.

### 4.5. Analysis of the mechanism of influence factors on coal power supply chain resilience

**Fig 6:**
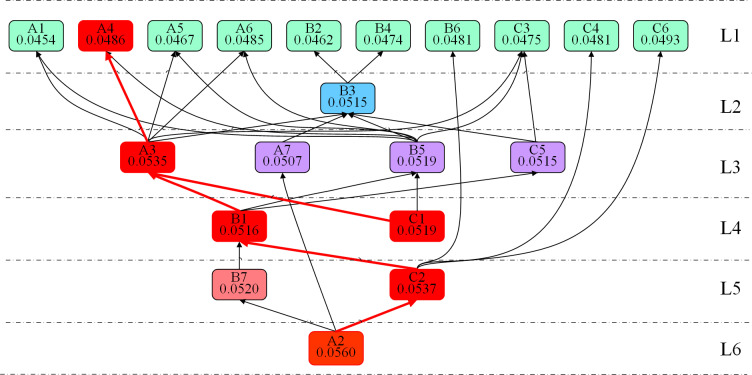
Pathways of influence on coal power supply chain resilience.

By integrating the hierarchical structure diagram derived from the DEMATEL-ISM combined model with the ANP, we can perform a comprehensive analysis to determine the weight of each influencing factor. These weights reflect the relative importance of each factor in shaping the resilience level of the supply chain. This combined approach allows for a more nuanced understanding of how different factors interact and contribute to the overall resilience, providing a quantitative basis for strategic decision-making aimed at strengthening the coal power supply chain’s ability to withstand and recover from disruptions. The pathways of influence on coal power supply chain resilience are shown in Fig 6.

The core driving factor of the coal power supply chain resilience system is Electricity Demand Forecasting Capability (A2), which resides at the lowest level of the hierarchy (L6) and possesses both high weight and causality. This underscores the critical importance of accurate electricity demand forecasting for the effective operation of the entire supply chain [72]. By accurately predicting future electricity demand, it provides data support for resource allocation and dispatch within the supply chain. This forecasting capability not only facilitates the dynamic balance of electricity supply and demand but also exerts a profound impact on higher-level dispatching and reserve decision-making. Changes in A2 are transmitted layer by layer through the intermediate factors of the coal power supply chain resilience system, significantly influencing upper-level decisions and operational approaches, thus becoming a key driver for the dynamic adjustment and optimization of the entire supply chain.

At the intermediate level, factors such as Technological Level of the Coal Industry (C2), Risk Perception Capability (B1), and Degree of Government Intervention in the Market (C1) play a key role in transmitting bottom-up driving forces to the upper levels. The efficiency and flexibility of the Technological Level of the Coal Industry (C2) directly influence the reliability of Coal Emergency Reserve Assurance Capability (A4), providing the necessary resources to support electricity demand forecasting and ensuring that the supply chain can swiftly allocate reserve resources in response to increased demand. Risk Perception Capability (B1) helps identify potential risks and promptly communicates early warning information to dispatch and prevention mechanisms, thereby enhancing the coal power supply chain’s response speed and emergency preparedness. The Degree of Government Intervention in the Market (C1) regulates corporate behavior and market activities through the formulation and implementation of policies, ensuring that the supply chain’s resources are reasonably allocated during market fluctuations or emergency situations, thereby providing policy support for the stability of the supply chain.

At the first and second layers of the coal power supply chain resilience system, factors such as Risk Prevention and Maintenance Level (A1), Grid Dispatching Capability (A3), and Electricity Storage Technology Level (A5) serve as the ultimate decision-making and emergency mechanisms, reflecting the coal power supply chain’s feedback control capacity in response to demand fluctuations. Risk Prevention and Maintenance Level (A1) ensures that the coal power supply chain can effectively cope with unforeseen events and maintain stable operation by proactively identifying potential risks and implementing maintenance measures. Grid Dispatching Capability (A3), based on bottom-layer electricity demand forecasting (A2) and the information transmitted from the intermediate layer, facilitates the dynamic allocation of electricity resources, enabling the power system to adjust in real-time according to actual demand. Electricity Storage Technology Level (A5) provides technical support for the storage and dispatch of electricity, ensuring that adequate power reserves are available to handle emergencies during peak demand periods.

Government intervention and market coordination mechanisms (A7 and C1) have further strengthened the emergency response and regulation capabilities of the coal power supply chain. Government Intervention and Coordination Capability (A7) ensures the efficient operation of the market in emergency situations by guiding policies and coordinating resource allocation. This mechanism achieves “policy-market” synergy by balancing supply and demand and directing resource flows, ensuring the rational allocation of resources within the coal power supply chain and enhancing its resilience to sudden events.

The Diversity of Enterprise Emergency Actions (B3) and the Relationship between Enterprises and Service Organizations (B4) provide support for the overall resilience of the coal power supply chain by improving enterprises’ response and coordination capabilities in emergencies. The Diversity of Enterprise Emergency Actions (B3) enables enterprises to flexibly adapt to changes in the external environment through diverse response strategies. The Relationship between Enterprises and Service Organizations (B4) enhances the overall emergency response capability of the coal power supply chain through cooperation with external support organizations. This collaboration facilitates rapid mobilization during emergencies, ensuring the timely supply of emergency resources.

Additionally, Information Transmission Efficiency (B5) and Technological Innovation Capability (C5) provide essential support within the coal power supply chain. Information Transmission Efficiency (B5) ensures the rapid flow of information across different levels, enabling efficient coordination throughout the supply chain. Technological Innovation Capability (C5) continuously introduces new technologies and methods into the supply chain, enhancing its emergency response and scheduling capabilities and ensuring the long-term development of the coal power supply chain.

## 5. Discussion

In the resilience management of the coal power supply chain, the integration of fuzzy DEMATEL, ISM, and ANP methods has enabled the identification of key influencing factors and analysis of their mechanisms of action. The analysis results indicate that Electricity Demand Forecasting Capability (A2) [73], Degree of Government Intervention in the Market (C1) [74], and Technological Level of the Coal Industry (C2) [75] are the primary driving factors for the resilience of the coal power supply chain. These factors significantly impact the stability and smooth operation of the supply chain, with Electricity Demand Forecasting Capability (A2) serving as the fundamental driving force that impoves proactive planning and rapid response to future demand. This conclusion is consistent with the study by Wu et al., which emphasizes the importance of forecasting capability in responding to external shocks in energy supply chains [76]. Similarly, Nyangon and Akintunde also demonstrated in their research that the accuracy of forecasting improves resource integration and contributes to better energy management [77]. Meanwhile, the enhancement of Government Intervention Capability (C1) and Technological Level of the Coal Industry (C2) ensures the rational allocation of resources through policy support and technological advancements.

The results of the ISM model further reveal the hierarchical relationships among these factors. Electricity Demand Forecasting Capability (A2), positioned at the fundamental level, is crucial for the overall stability of the coal power supply chain. Meanwhile, the study by Nyangon and Byrne also pointed out that market forecasting and flexible scheduling are crucial for the stability of the power supply chain [78]. In the intermediate layer, Government Intervention and Coordination Capability (A7), Grid Dispatching Capability (A3), and Information Transmission Efficiency (B5) serve as key supporting elements, enhancing the efficient coordination and resource allocation across various stages of the supply chain. Similarly, Chang and Jiang studied how government policies affect the resilience of the food supply chain, highlighting that policy intervention and the efficiency of information flow play crucial roles in supply chain resilience [79]. Likewise, Nyangon et al. mentioned in their research that the policy environment helps solar photovoltaic technology maintain market share even under low natural gas prices, demonstrating the significant role of government intervention in stabilizing the market [80]. The Diversity of Enterprise Emergency Actions (B3), as an important linking factor, provides the coal power supply chain with flexible response capabilities in emergency management. At the first layer, Coal Emergency Reserve Assurance Capability (A4) and Electricity Storage Technology Level (A5) play significant roles in recovery capacity during unforeseen events, ensuring the resilience of the supply chain in crisis situations.

The weight analysis of the ANP model reveals that Electricity Demand Forecasting Capability (A2) holds the highest weight (0.0560), underscoring its critical role in the proactive planning of the coal power supply chain. Following this, the Technological Level of the Coal Industry (C2) and Grid Dispatching Capability (A3) emerge as important factors, highlighting the significance of technological upgrades and resource scheduling in enhancing the resilience of the coal power supply chain. Additionally, Information Transmission Efficiency (B5) and Government Intervention Capability (C1) also carry relatively high weights, indicating that optimizing information flow and policy regulation plays a crucial role in maintaining the stability of the coal power supply chain. In line with this, Taminiau et al. proposed strategies for urban energy transition, emphasizing the crucial role of government policies and technological development in the transformation of energy systems [81].

Through the mechanism analysis, it is evident that Electricity Demand Forecasting Capability (A2) enhances the Technological Level of the Coal Industry (C2), which, in turn, further strengthens Grid Dispatching Capability (A3), thereby enabling the efficient allocation of energy resources. This pathway highlights the close interconnection between demand forecasting, technological upgrades, and resource scheduling. Furthermore, the interaction between Government Intervention Capability (C1) and Grid Dispatching Capability (A3) indicates that governmental policy support during market fluctuations is crucial for ensuring the smooth operation of the supply chain.

In summary, the resilience management of the coal power supply chain should prioritize the enhancement of Electricity Demand Forecasting Capability (A2) by developing an intelligent forecasting system to ensure the precision of resource allocation. Additionally, improving the Technological Level of the Coal Industry (C2) will enhance market adaptability and production efficiency. Optimizing Grid Dispatching Capability (A3) will strengthen the supply chain’s rapid response capacity during unforeseen events, while enhancing Information Transmission Efficiency (B5) and Government Intervention Capability (C1) will ensure the efficient operation and adaptability of the coal power supply chain. Through the collaborative optimization of these key pathways, the coal power supply chain will develop stronger resilience and risk resistance, providing a solid foundation for long-term stable development in a complex market environment.

Based on the above analysis, we propose the following strategies. First, enhancing Electricity Demand Forecasting Capability is crucial [73]. Developing intelligent forecasting systems and leveraging big data analysis can strengthen the proactive planning capability of the coal power supply chain, enabling effective responses to future supply-demand fluctuations [82]. Accurate electricity demand forecasting not only facilitates scheduling and planning but also helps mitigate issues of waste or shortages caused by supply-demand mismatches, which is critical for ensuring the stability of power supply.

Secondly, promoting technological upgrades in the coal industry is also a critical means of enhancing supply chain resilience. According to research, green technology innovation not only improves environmental performance but also drives technological progress in the industry through government policy support, thereby enhancing market competitiveness and production efficiency [83]. Therefore, the coal industry should strengthen research and development in green technologies and optimize production processes to improve the resilience of the coal power supply chain and better address the challenges of energy transition.

Additionally, optimizing grid dispatching capability and information transmission efficiency are also crucial for enhancing the supply chain’s resilience to risks. By establishing advanced IoT devices and information systems, real-time emergency data can be collected and analyzed, thereby improving the enterprise’s emergency response capacity and flexibility [84]. An effective information transmission system helps to quickly coordinate resources across all parties, ensuring the stable operation of the coal power supply chain.

Finally, government policy support is crucial. The government needs to play an active role in coordinating and intervening in the market by formulating flexible policies that ensure the rational allocation of resources. For instance, by enacting favorable regulations and policies, the government can encourage collaboration and technological innovation among the parties in the coal power supply chain, further enhancing the overall resilience and adaptability of the coal power supply chain.

## 6. Conclusion

### 6.1. summary

The contribution of this paper lies in systematically identifying 20 influencing factors of coal power supply chain resilience, establishing a framework for these factors, and integrating fuzzy DEMATEL, ISM, and ANP methods. The main findings of this paper are as follows:

(1) The results show that Electricity Demand Forecasting Capability, Government Intervention Capability, and Technological Level of the Coal Industry are the key driving factors, determining the planning, operation, and market regulation of the coal power supply chain.
(2) The analysis of the mechanism of action reveals that Electricity Demand Forecasting Capability, as the fundamental driving factor of coal power supply chain resilience, provides the essential foundation for the rational allocation of resources through accurate demand forecasting. The impact of Electricity Demand Forecasting Capability is further transmitted through the Technological Level of the Coal Industry to Grid Dispatching Capability, forming the primary pathway: Electricity Demand Forecasting Capability → Technological Level of the Coal Industry → Risk Perception Capability → Grid Dispatching Capability. Coal Emergency Reserve Assurance Capability is a key part of this pathway, illustrating how demand forecasting drives technological upgrades and, through efficient dispatch, achieves resource optimization. Furthermore, the pathway Government Intervention Capability → Grid Dispatching Capability → Coal Emergency Reserve Assurance Capability emphasizes the critical role of government intervention in emergency management.

### 6.2. Implications

This study provides a comprehensive theoretical framework for the coal power industry, systematically analyzing the impact of factors such as electricity demand forecasting capability, the technological level of the coal industry, and government intervention on supply chain resilience. This framework helps coal power enterprises understand the multi-dimensional factors affecting supply chain resilience from a global perspective and offers theoretical support for decision-makers within the industry to optimize supply chain management.

Through the integration of fuzzy DEMATEL, ISM, and ANP methods, this study offers a multi-perspective, comprehensive approach for identifying and quantifying the key factors influencing supply chain resilience. This method not only enhances the understanding of the interactions between different factors for coal power enterprises but also provides strong empirical support for how to improve supply chain resilience in practical operations.

The study presents a series of specific policy and technological recommendations, including strengthening electricity demand forecasting capabilities, promoting green technological innovation in the coal industry, and optimizing the power grid scheduling system. These recommendations provide practical and feasible strategies for coal power enterprises to respond to the increasingly complex energy environment, policy changes, and market demand fluctuations.

### 6.3. Research limitations

This study has certain limitations. First, due to subjective constraints, it was challenging to construct a comprehensive, scientific, and objective resilience indicator system when selecting the metrics, and the range of influencing factors selected remains limited. Future research could utilize artificial intelligence recommendation systems to screen and identify indicators with objectivity and high efficiency. Additionally, optimizing and expanding the analysis and transformation process could allow intelligent systems to select more relevant and impactful fundamental factors, thereby improving the effectiveness and scientific rigor of resilience strategies. Second, with the development of new energy and technological advancements, the resilience of the coal power supply chain may face new challenges and opportunities. Therefore, future studies could further explore how to optimize the resilience of the coal power supply chain in the context of rapid new energy development, especially in balancing the supply chain coordination between traditional coal power and renewable energy during the energy transition process. Additionally, the analysis in this study is primarily based on theoretical and model construction, without empirical research being considered. Future research could incorporate field surveys, case studies, or big data analysis for empirical validation, ensuring that the conclusions are more applicable and valuable in real-world scenarios.

## Supporting information

S1 File F-DEMATEL-ISM-ANP Procedure. This file displays the results obtained using the Fuzzy DEMATEL-ISM-ANP approach.
(DOCX)
