# Supplementary material for: Resilience factors and mechanisms in the coal power supply chain: A quantitative analysis using fuzzy DEMATEL, ISM, and ANP methods
Source: PLoS One. 2025 Jun 2;20(6):e0322952. doi: 10.1371/journal.pone.0322952 (PMC12129186; doi:10.1371/journal.pone.0322952)
Supplement: S1 File — This file displays the results obtained using the Fuzzy DEMATEL-ISM-ANP approach. (DOCX) [file pone.0322952.s001.docx]

**S1 FILE: SUPPORTING INFORMATION**

**A. Step by step execution of the F-DEMATEL procedure**

**Step 1: Determining of influencing factors in the system**

***Table S1*** below shows the factors used in this study after the literature survey.

**Table S1: The 20 Factors Used in the Study**

| Primary Indicators | Secondary Indicators | Description |
| --- | --- | --- |
| Restorative Capacity | Risk Prevention and Maintenance Level (A1) | Preventive measures taken by enterprises and the government to address supply chain disruption risks, including equipment maintenance, transportation management, and disaster prevention. |
|  | Electricity Demand Forecasting Capability (A2) | The ability of power companies to accurately forecast demand based on historical data and market changes to ensure a balance between production and consumption. |
|  | Grid Dispatching Capability (A3) | The ability of the power dispatch center to allocate power resources during demand fluctuations to ensure stable power supply. |
|  | Coal Emergency Reserve Assurance Capability (A4) | The capability to efficiently utilize emergency coal reserves during critical moments, including logistics and reserve facilities. |
|  | Electricity Storage Technology Level (A5) | Efficient electricity storage technologies that help balance grid supply and demand during demand fluctuations. |
|  | Electricity Emergency Dispatch System (A6) | The emergency dispatch system of the grid in response to sudden incidents, including the coordinated dispatch of various power production units. |
|  | Government Intervention and Coordination Capability (A7) | The ability of the government to coordinate and allocate the market in emergencies, ensuring stable supply chain operations. |
| Absorptive Capacity | Risk Perception Capability (B1) | The ability of enterprises to perceive and predict market fluctuations, energy price changes, and potential supply disruptions. |
|  | Non-State Economic Development Level (B2) | The extent and depth of participation of non-state economies in the coal power supply chain, increasing market diversity and resilience. |
|  | Diversity of Enterprise Emergency Actions (B3) | The variety of emergency measures taken by enterprises during supply chain disruptions. |
|  | Relationship between Enterprises and Service Organizations (B4) | The impact of cooperation between enterprises and logistics, technical services, and other key service organizations on supply chain resilience. |
|  | Information Transmission Efficiency (B5) | The speed and accuracy of information transmission between nodes in the supply chain. |
|  | Asset Liquidity (B6) | The liquidity and flexibility of enterprise assets to quickly allocate resources to respond to emergencies in the face of risks or fluctuations. |
|  | Relevant Legal and Policy Level (B7) | The impact of changes in government policies and regulations related to the coal and power industries on supply chain management. |
| Adaptive Capacity | Degree of Government Intervention in the Market (C1) | The extent of government regulation in the coal power supply chain, such as market regulation and price intervention measures. |
|  | Technological Level of the Coal Industry (C2) | The support provided by advanced coal production technology to supply chain resilience. |
|  | Development Level of Service Organizations (C3) | The level of support and assurance provided by third-party services related to the supply chain. |
|  | Industry Resource Competitiveness (C4) | The competitive advantage of the coal power industry in regional markets. |
|  | Technological Innovation Capability (C5) | The capability of enterprises to innovate technology within the coal power industry. |
|  | Product Market Development Level (C6) | The maturity and development level of coal and related energy product markets. |

This table lists the 20 factors selected in this study that affect the resilience of the coal power supply chain, along with a description of each factor.

**Step 2: Designing the fuzzy linguistic scale**

The fuzzy linguistic scale in ***Table S2*** was used to collect feedback from the experts.

**Table S2: Influencing Factors and Expert Semantic Conversion Table**

| Semantic Variable | Expert Score | Triangular Fuzzy Number (TFN) |
| --- | --- | --- |
| No Influence（N） | 0 | (0.00 ,0.00 ,0.25) |
| Low Influence（L） | 1 | (0.00, 0.25, 0.50) |
| Moderate Influence（M） | 2 | (0.25, 0.50, 0.75) |
| High Influence（H） | 3 | (0.50, 0.75, 1.00) |
| Very High Influence（VH） | 4 | (0.75, 1.00, 1.00) |

This table shows the defuzzified direct relation matrix derived from expert ratings, reflecting the mutual influences among the factors.

**Step 3: Constructing the direct relation matrix**

The triangular fuzzy number is used to represent the influence relationship among various factors, and then the direct influence matrix Z obtained by defuzzification method, as shown in ***Table S3***.

**Table S3: Defuzzifying Initial Direct Relation Matrix**

|  | **A1** | **A2** | **A3** | **A4** | **A5** | **A6** | **A7** | **B1** | **B2** | **B3** | **B4** | **B5** | **B6** | **B7** | **C1** | **C2** | **C3** | **C4** | **C5** | **C6** |
| --- | --- | --- | --- | --- | --- | --- | --- | --- | --- | --- | --- | --- | --- | --- | --- | --- | --- | --- | --- | --- |
| **A1** | **0.0417** | **0.5000** | **0.5000** | **0.6167** | **0.5292** | **0.6167** | **0.6750** | **0.4417** | **0.4125** | **0.5875** | **0.5583** | **0.7333** | **0.2958** | **0.4417** | **0.5583** | **0.5292** | **0.4708** | **0.3250** | **0.5875** | **0.3250** |
| **A2** | **0.6167** | **0.0417** | **0.6750** | **0.7042** | **0.5000** | **0.5875** | **0.5875** | **0.8792** | **0.3833** | **0.7333** | **0.6458** | **0.5000** | **0.6750** | **0.6458** | **0.5875** | **0.7042** | **0.7042** | **0.4125** | **0.6458** | **0.9375** |
| **A3** | **0.7625** | **0.5583** | **0.0417** | **0.7625** | **0.7042** | **0.7625** | **0.5875** | **0.4417** | **0.4125** | **0.8792** | **0.6750** | **0.7917** | **0.3833** | **0.7042** | **0.7042** | **0.4708** | **0.6750** | **0.5583** | **0.3542** | **0.4125** |
| **A4** | **0.5875** | **0.1792** | **0.5875** | **0.0417** | **0.5000** | **0.5583** | **0.5583** | **0.6167** | **0.6167** | **0.5875** | **0.5000** | **0.3833** | **0.5583** | **0.5875** | **0.4708** | **0.7625** | **0.7333** | **0.5875** | **0.5292** | **0.6167** |
| **A5** | **0.4125** | **0.6167** | **0.7042** | **0.5000** | **0.0417** | **0.5583** | **0.4708** | **0.5875** | **0.5583** | **0.5583** | **0.5000** | **0.3833** | **0.6750** | **0.4417** | **0.2375** | **0.5583** | **0.5583** | **0.4708** | **0.6458** | **0.5875** |
| **A6** | **0.5000** | **0.5875** | **0.6750** | **0.3542** | **0.3833** | **0.0417** | **0.4708** | **0.6750** | **0.7042** | **0.5583** | **0.3542** | **0.5875** | **0.7625** | **0.4125** | **0.4417** | **0.5000** | **0.5292** | **0.6167** | **0.5875** | **0.7333** |
| **A7** | **0.7333** | **0.5292** | **0.6458** | **0.6750** | **0.7333** | **0.5875** | **0.0417** | **0.4417** | **0.6458** | **0.6458** | **0.4708** | **0.6167** | **0.4417** | **0.5583** | **0.6458** | **0.5292** | **0.5583** | **0.4417** | **0.6167** | **0.4417** |
| **B1** | **0.5000** | **0.4708** | **0.3833** | **0.8792** | **0.6458** | **0.6167** | **0.5000** | **0.0417** | **0.7625** | **0.6750** | **0.7333** | **0.6458** | **0.6458** | **0.4708** | **0.5292** | **0.3542** | **0.4708** | **0.5583** | **0.7333** | **0.6458** |
| **B2** | **0.5875** | **0.5583** | **0.6458** | **0.6750** | **0.3542** | **0.2667** | **0.3542** | **0.5292** | **0.0417** | **0.2667** | **0.3542** | **0.5000** | **0.2958** | **0.5000** | **0.7333** | **0.5292** | **0.4708** | **0.7333** | **0.7917** | **0.7333** |
| **B3** | **0.6750** | **0.5875** | **0.5583** | **0.6458** | **0.4708** | **0.6167** | **0.5292** | **0.6458** | **0.7333** | **0.0417** | **0.8208** | **0.5875** | **0.5875** | **0.6167** | **0.3542** | **0.4417** | **0.5583** | **0.4417** | **0.7042** | **0.5875** |
| **B4** | **0.6458** | **0.5000** | **0.6750** | **0.5875** | **0.4417** | **0.4708** | **0.5583** | **0.5000** | **0.5583** | **0.5583** | **0.0417** | **0.6167** | **0.5875** | **0.5583** | **0.3250** | **0.6458** | **0.4417** | **0.6458** | **0.4708** | **0.4125** |
| **B5** | **0.7625** | **0.6167** | **0.6458** | **0.3250** | **0.5583** | **0.7042** | **0.5292** | **0.6167** | **0.5583** | **0.8208** | **0.5000** | **0.0417** | **0.6458** | **0.6750** | **0.5583** | **0.6458** | **0.7042** | **0.3542** | **0.5292** | **0.4417** |
| **B6** | **0.4125** | **0.5292** | **0.6458** | **0.4417** | **0.5000** | **0.5000** | **0.7042** | **0.5292** | **0.5000** | **0.3833** | **0.6167** | **0.7042** | **0.0417** | **0.5292** | **0.3833** | **0.6458** | **0.5875** | **0.5000** | **0.5875** | **0.6167** |
| **B7** | **0.6750** | **0.3833** | **0.3542** | **0.5000** | **0.6167** | **0.6167** | **0.7625** | **0.7042** | **0.5000** | **0.5583** | **0.7042** | **0.6750** | **0.5875** | **0.0417** | **0.6458** | **0.5292** | **0.7625** | **0.4708** | **0.6167** | **0.6167** |
| **C1** | **0.7042** | **0.6750** | **0.6167** | **0.6750** | **0.4708** | **0.6167** | **0.5583** | **0.5875** | **0.4417** | **0.5875** | **0.7625** | **0.5583** | **0.6750** | **0.6167** | **0.0417** | **0.5000** | **0.4417** | **0.6750** | **0.5000** | **0.5583** |
| **C2** | **0.4708** | **0.7333** | **0.5583** | **0.4125** | **0.7917** | **0.5292** | **0.5875** | **0.6167** | **0.6458** | **0.7042** | **0.6167** | **0.6750** | **0.6750** | **0.4708** | **0.6458** | **0.0417** | **0.5292** | **0.6458** | **0.7042** | **0.5875** |
| **C3** | **0.4125** | **0.4708** | **0.5875** | **0.5292** | **0.7042** | **0.5000** | **0.4417** | **0.3250** | **0.6750** | **0.3833** | **0.6750** | **0.6750** | **0.5875** | **0.4417** | **0.4125** | **0.5583** | **0.0417** | **0.8500** | **0.5292** | **0.5000** |
| **C4** | **0.4708** | **0.5000** | **0.5292** | **0.4125** | **0.6750** | **0.5583** | **0.3250** | **0.7042** | **0.7625** | **0.6750** | **0.5000** | **0.4417** | **0.4125** | **0.4125** | **0.6750** | **0.5292** | **0.6167** | **0.0417** | **0.3833** | **0.7917** |
| **C5** | **0.4125** | **0.4125** | **0.5875** | **0.4417** | **0.5000** | **0.4125** | **0.5875** | **0.6167** | **0.6750** | **0.7333** | **0.7333** | **0.5875** | **0.6167** | **0.7042** | **0.5875** | **0.5000** | **0.7625** | **0.7917** | **0.0417** | **0.4708** |
| **C6** | **0.6458** | **0.5875** | **0.6750** | **0.4417** | **0.5875** | **0.5583** | **0.6750** | **0.6167** | **0.5292** | **0.5292** | **0.4125** | **0.5292** | **0.5292** | **0.6167** | **0.4708** | **0.5875** | **0.6167** | **0.5875** | **0.4125** | **0.0417** |

This table shows the defuzzified direct relation matrix derived from expert ratings, reflecting the mutual influences among the factors.

**Step 4: Normalizing the direct relation fuzzy matrix**

The normalized direct relation matrix was normalized by dividing each of the elements of the initial direct relation matrix by the maximum value of the sums of the rows and columns of the initial direct relation matrix (Equation 1), and the results are shown in ***Table S4***.

**Table S4: Normalized Direct Relation Matrix**

|  | **A1** | **A2** | **A3** | **A4** | **A5** | **A6** | **A7** | **B1** | **B2** | **B3** | **B4** | **B5** | **B6** | **B7** | **C1** | **C2** | **C3** | **C4** | **C5** | **C6** |
| --- | --- | --- | --- | --- | --- | --- | --- | --- | --- | --- | --- | --- | --- | --- | --- | --- | --- | --- | --- | --- |
| **A1** | **0.0034** | **0.0411** | **0.0411** | **0.0507** | **0.0435** | **0.0507** | **0.0555** | **0.0363** | **0.0339** | **0.0483** | **0.0459** | **0.0603** | **0.0243** | **0.0363** | **0.0459** | **0.0435** | **0.0387** | **0.0267** | **0.0483** | **0.0267** |
| **A2** | **0.0507** | **0.0034** | **0.0555** | **0.0579** | **0.0411** | **0.0483** | **0.0483** | **0.0723** | **0.0315** | **0.0603** | **0.0531** | **0.0411** | **0.0555** | **0.0531** | **0.0483** | **0.0579** | **0.0579** | **0.0339** | **0.0531** | **0.0771** |
| **A3** | **0.0627** | **0.0459** | **0.0034** | **0.0627** | **0.0579** | **0.0627** | **0.0483** | **0.0363** | **0.0339** | **0.0723** | **0.0555** | **0.0651** | **0.0315** | **0.0579** | **0.0579** | **0.0387** | **0.0555** | **0.0459** | **0.0291** | **0.0339** |
| **A4** | **0.0483** | **0.0147** | **0.0483** | **0.0034** | **0.0411** | **0.0459** | **0.0459** | **0.0507** | **0.0507** | **0.0483** | **0.0411** | **0.0315** | **0.0459** | **0.0483** | **0.0387** | **0.0627** | **0.0603** | **0.0483** | **0.0435** | **0.0507** |
| **A5** | **0.0339** | **0.0507** | **0.0579** | **0.0411** | **0.0034** | **0.0459** | **0.0387** | **0.0483** | **0.0459** | **0.0459** | **0.0411** | **0.0315** | **0.0555** | **0.0363** | **0.0195** | **0.0459** | **0.0459** | **0.0387** | **0.0531** | **0.0483** |
| **A6** | **0.0411** | **0.0483** | **0.0555** | **0.0291** | **0.0315** | **0.0034** | **0.0387** | **0.0555** | **0.0579** | **0.0459** | **0.0291** | **0.0483** | **0.0627** | **0.0339** | **0.0363** | **0.0411** | **0.0435** | **0.0507** | **0.0483** | **0.0603** |
| **A7** | **0.0603** | **0.0435** | **0.0531** | **0.0555** | **0.0603** | **0.0483** | **0.0034** | **0.0363** | **0.0531** | **0.0531** | **0.0387** | **0.0507** | **0.0363** | **0.0459** | **0.0531** | **0.0435** | **0.0459** | **0.0363** | **0.0507** | **0.0363** |
| **B1** | **0.0411** | **0.0387** | **0.0315** | **0.0723** | **0.0531** | **0.0507** | **0.0411** | **0.0034** | **0.0627** | **0.0555** | **0.0603** | **0.0531** | **0.0531** | **0.0387** | **0.0435** | **0.0291** | **0.0387** | **0.0459** | **0.0603** | **0.0531** |
| **B2** | **0.0483** | **0.0459** | **0.0531** | **0.0555** | **0.0291** | **0.0219** | **0.0291** | **0.0435** | **0.0034** | **0.0219** | **0.0291** | **0.0411** | **0.0243** | **0.0411** | **0.0603** | **0.0435** | **0.0387** | **0.0603** | **0.0651** | **0.0603** |
| **B3** | **0.0555** | **0.0483** | **0.0459** | **0.0531** | **0.0387** | **0.0507** | **0.0435** | **0.0531** | **0.0603** | **0.0034** | **0.0675** | **0.0483** | **0.0483** | **0.0507** | **0.0291** | **0.0363** | **0.0459** | **0.0363** | **0.0579** | **0.0483** |
| **B4** | **0.0531** | **0.0411** | **0.0555** | **0.0483** | **0.0363** | **0.0387** | **0.0459** | **0.0411** | **0.0459** | **0.0459** | **0.0034** | **0.0507** | **0.0483** | **0.0459** | **0.0267** | **0.0531** | **0.0363** | **0.0531** | **0.0387** | **0.0339** |
| **B5** | **0.0627** | **0.0507** | **0.0531** | **0.0267** | **0.0459** | **0.0579** | **0.0435** | **0.0507** | **0.0459** | **0.0675** | **0.0411** | **0.0034** | **0.0531** | **0.0555** | **0.0459** | **0.0531** | **0.0579** | **0.0291** | **0.0435** | **0.0363** |
| **B6** | **0.0339** | **0.0435** | **0.0531** | **0.0363** | **0.0411** | **0.0411** | **0.0579** | **0.0435** | **0.0411** | **0.0315** | **0.0507** | **0.0579** | **0.0034** | **0.0435** | **0.0315** | **0.0531** | **0.0483** | **0.0411** | **0.0483** | **0.0507** |
| **B7** | **0.0555** | **0.0315** | **0.0291** | **0.0411** | **0.0507** | **0.0507** | **0.0627** | **0.0579** | **0.0411** | **0.0459** | **0.0579** | **0.0555** | **0.0483** | **0.0034** | **0.0531** | **0.0435** | **0.0627** | **0.0387** | **0.0507** | **0.0507** |
| **C1** | **0.0579** | **0.0555** | **0.0507** | **0.0555** | **0.0387** | **0.0507** | **0.0459** | **0.0483** | **0.0363** | **0.0483** | **0.0627** | **0.0459** | **0.0555** | **0.0507** | **0.0034** | **0.0411** | **0.0363** | **0.0555** | **0.0411** | **0.0459** |
| **C2** | **0.0387** | **0.0603** | **0.0459** | **0.0339** | **0.0651** | **0.0435** | **0.0483** | **0.0507** | **0.0531** | **0.0579** | **0.0507** | **0.0555** | **0.0555** | **0.0387** | **0.0531** | **0.0034** | **0.0435** | **0.0531** | **0.0579** | **0.0483** |
| **C3** | **0.0339** | **0.0387** | **0.0483** | **0.0435** | **0.0579** | **0.0411** | **0.0363** | **0.0267** | **0.0555** | **0.0315** | **0.0555** | **0.0555** | **0.0483** | **0.0363** | **0.0339** | **0.0459** | **0.0034** | **0.0699** | **0.0435** | **0.0411** |
| **C4** | **0.0387** | **0.0411** | **0.0435** | **0.0339** | **0.0555** | **0.0459** | **0.0267** | **0.0579** | **0.0627** | **0.0555** | **0.0411** | **0.0363** | **0.0339** | **0.0339** | **0.0555** | **0.0435** | **0.0507** | **0.0034** | **0.0315** | **0.0651** |
| **C5** | **0.0339** | **0.0339** | **0.0483** | **0.0363** | **0.0411** | **0.0339** | **0.0483** | **0.0507** | **0.0555** | **0.0603** | **0.0603** | **0.0483** | **0.0507** | **0.0579** | **0.0483** | **0.0411** | **0.0627** | **0.0651** | **0.0034** | **0.0387** |
| **C6** | **0.0531** | **0.0483** | **0.0555** | **0.0363** | **0.0483** | **0.0459** | **0.0555** | **0.0507** | **0.0435** | **0.0435** | **0.0339** | **0.0435** | **0.0435** | **0.0507** | **0.0387** | **0.0483** | **0.0507** | **0.0483** | **0.0339** | **0.0034** |

This table presents the normalized direct relation matrix, which reflects the relative impact degree among the factors.

**Step 5: Calculating the comprehenxive influence matrix using Equation 2, and the calculation results are shown in *Table S5*.**

**Table S5: The comprehensive influence matrix**

|  | **A1** | **A2** | **A3** | **A4** | **A5** | **A6** | **A7** | **B1** | **B2** | **B3** | **B4** | **B5** | **B6** | **B7** | **C1** | **C2** | **C3** | **C4** | **C5** | **C6** |
| --- | --- | --- | --- | --- | --- | --- | --- | --- | --- | --- | --- | --- | --- | --- | --- | --- | --- | --- | --- | --- |
| **A1** | **0.3371** | **0.3439** | **0.3805** | **0.3698** | **0.3655** | **0.3718** | **0.3704** | **0.3698** | **0.3694** | **0.3927** | **0.3814** | **0.3968** | **0.3469** | **0.3519** | **0.3470** | **0.3611** | **0.3764** | **0.3501** | **0.3778** | **0.3584** |
| **A2** | **0.4607** | **0.3795** | **0.4739** | **0.4531** | **0.4407** | **0.4462** | **0.4399** | **0.4821** | **0.4471** | **0.4850** | **0.4687** | **0.4603** | **0.4520** | **0.4422** | **0.4207** | **0.4497** | **0.4744** | **0.4342** | **0.4604** | **0.4839** |
| **A3** | **0.4540** | **0.4034** | **0.4058** | **0.4390** | **0.4373** | **0.4419** | **0.4214** | **0.4307** | **0.4304** | **0.4769** | **0.4516** | **0.4627** | **0.4120** | **0.4287** | **0.4124** | **0.4147** | **0.4532** | **0.4260** | **0.4200** | **0.4257** |
| **A4** | **0.4034** | **0.3420** | **0.4115** | **0.3481** | **0.3879** | **0.3904** | **0.3847** | **0.4068** | **0.4103** | **0.4167** | **0.4017** | **0.3958** | **0.3895** | **0.3852** | **0.3630** | **0.4016** | **0.4207** | **0.3950** | **0.3976** | **0.4052** |
| **A5** | **0.3757** | **0.3618** | **0.4061** | **0.3709** | **0.3371** | **0.3765** | **0.3646** | **0.3910** | **0.3907** | **0.4002** | **0.3872** | **0.3809** | **0.3848** | **0.3612** | **0.3319** | **0.3728** | **0.3935** | **0.3716** | **0.3921** | **0.3895** |
| **A6** | **0.3961** | **0.3723** | **0.4174** | **0.3726** | **0.3773** | **0.3488** | **0.3771** | **0.4110** | **0.4150** | **0.4139** | **0.3895** | **0.4100** | **0.4039** | **0.3717** | **0.3602** | **0.3808** | **0.4046** | **0.3954** | **0.4008** | **0.4139** |
| **A7** | **0.4303** | **0.3822** | **0.4319** | **0.4126** | **0.4193** | **0.4077** | **0.3585** | **0.4095** | **0.4266** | **0.4375** | **0.4149** | **0.4283** | **0.3956** | **0.3981** | **0.3898** | **0.3989** | **0.4233** | **0.3976** | **0.4194** | **0.4071** |
| **B1** | **0.4194** | **0.3835** | **0.4196** | **0.4344** | **0.4187** | **0.4159** | **0.4013** | **0.3850** | **0.4434** | **0.4462** | **0.4413** | **0.4369** | **0.4180** | **0.3983** | **0.3867** | **0.3929** | **0.4239** | **0.4139** | **0.4352** | **0.4302** |
| **B2** | **0.3855** | **0.3532** | **0.3970** | **0.3801** | **0.3585** | **0.3507** | **0.3518** | **0.3827** | **0.3452** | **0.3747** | **0.3722** | **0.3849** | **0.3515** | **0.3621** | **0.3671** | **0.3667** | **0.3829** | **0.3879** | **0.3980** | **0.3960** |
| **B3** | **0.4318** | **0.3916** | **0.4314** | **0.4163** | **0.4050** | **0.4152** | **0.4031** | **0.4310** | **0.4397** | **0.3961** | **0.4472** | **0.4326** | **0.4123** | **0.4083** | **0.3732** | **0.3983** | **0.4295** | **0.4039** | **0.4321** | **0.4244** |
| **B4** | **0.3995** | **0.3579** | **0.4090** | **0.3824** | **0.3744** | **0.3755** | **0.3763** | **0.3897** | **0.3960** | **0.4062** | **0.3562** | **0.4041** | **0.3828** | **0.3750** | **0.3441** | **0.3847** | **0.3900** | **0.3892** | **0.3841** | **0.3810** |
| **B5** | **0.4409** | **0.3973** | **0.4403** | **0.3946** | **0.4147** | **0.4251** | **0.4057** | **0.4313** | **0.4289** | **0.4594** | **0.4266** | **0.3930** | **0.4197** | **0.4148** | **0.3908** | **0.4155** | **0.4427** | **0.3992** | **0.4218** | **0.4159** |
| **B6** | **0.3865** | **0.3646** | **0.4119** | **0.3755** | **0.3836** | **0.3821** | **0.3918** | **0.3963** | **0.3962** | **0.3978** | **0.4058** | **0.4154** | **0.3449** | **0.3774** | **0.3524** | **0.3891** | **0.4058** | **0.3832** | **0.3972** | **0.4008** |
| **B7** | **0.4351** | **0.3800** | **0.4196** | **0.4083** | **0.4202** | **0.4189** | **0.4239** | **0.4384** | **0.4261** | **0.4403** | **0.4422** | **0.4428** | **0.4163** | **0.3660** | **0.3979** | **0.4079** | **0.4478** | **0.4095** | **0.4289** | **0.4294** |
| **C1** | **0.4372** | **0.4012** | **0.4387** | **0.4213** | **0.4083** | **0.4189** | **0.4083** | **0.4300** | **0.4204** | **0.4427** | **0.4461** | **0.4333** | **0.4220** | **0.4110** | **0.3505** | **0.4057** | **0.4236** | **0.4236** | **0.4189** | **0.4254** |
| **C2** | **0.4320** | **0.4184** | **0.4480** | **0.4138** | **0.4450** | **0.4242** | **0.4221** | **0.4453** | **0.4491** | **0.4647** | **0.4483** | **0.4548** | **0.4350** | **0.4124** | **0.4094** | **0.3813** | **0.4433** | **0.4345** | **0.4478** | **0.4409** |
| **C3** | **0.3817** | **0.3564** | **0.4036** | **0.3775** | **0.3944** | **0.3775** | **0.3671** | **0.3770** | **0.4053** | **0.3929** | **0.4054** | **0.4080** | **0.3834** | **0.3663** | **0.3506** | **0.3788** | **0.3585** | **0.4060** | **0.3884** | **0.3886** |
| **C4** | **0.3909** | **0.3632** | **0.4034** | **0.3746** | **0.3961** | **0.3864** | **0.3627** | **0.4102** | **0.4166** | **0.4189** | **0.3974** | **0.3951** | **0.3749** | **0.3683** | **0.3742** | **0.3800** | **0.4073** | **0.3478** | **0.3826** | **0.4155** |
| **C5** | **0.4116** | **0.3786** | **0.4328** | **0.4005** | **0.4077** | **0.3996** | **0.4064** | **0.4282** | **0.4355** | **0.4494** | **0.4410** | **0.4322** | **0.4141** | **0.4143** | **0.3907** | **0.4018** | **0.4443** | **0.4304** | **0.3796** | **0.4154** |
| **C6** | **0.4130** | **0.3773** | **0.4229** | **0.3849** | **0.3989** | **0.3955** | **0.3981** | **0.4120** | **0.4074** | **0.4178** | **0.3997** | **0.4114** | **0.3918** | **0.3921** | **0.3673** | **0.3929** | **0.4168** | **0.3981** | **0.3933** | **0.3651** |

**Step 6: Calculate the impact and impact of each factor**

By summing the elements of the comprehensive influence matrix row by row, the influence degree of the corresponding factors is obtained. The results are shown in ***Table S6***.

**Table S6: Comprehensive impact table of factors affecting coal power supply chain resilience**

| **Factor** | **Influence**  **degree** | **Influenced**  **degree** | **Centrality**  **degree** | **Centrality**  **ranking** | **Cause**  **degree** |
| --- | --- | --- | --- | --- | --- |
| **A1** | **7.3186** | **8.2223** | **15.5410** | **19** | **-0.9037** |
| **A2** | **9.0547** | **7.5083** | **16.5630** | **5** | **1.5465** |
| **A3** | **8.6479** | **8.4053** | **17.0532** | **1** | **0.2425** |
| **A4** | **7.8572** | **7.9303** | **15.7875** | **15** | **-0.0732** |
| **A5** | **7.5402** | **7.9907** | **15.5309** | **20** | **-0.4505** |
| **A6** | **7.8322** | **7.9688** | **15.8011** | **14** | **-0.1366** |
| **A7** | **8.1893** | **7.8353** | **16.0245** | **11** | **0.3540** |
| **B1** | **8.3448** | **8.2581** | **16.6029** | **4** | **0.0867** |
| **B2** | **7.4488** | **8.2994** | **15.7482** | **17** | **-0.8506** |
| **B3** | **8.3231** | **8.5300** | **16.8531** | **2** | **-0.2069** |
| **B4** | **7.6581** | **8.3244** | **15.9824** | **12** | **-0.6663** |
| **B5** | **8.3783** | **8.3795** | **16.7578** | **3** | **-0.0012** |
| **B6** | **7.7583** | **7.9514** | **15.7097** | **18** | **-0.1932** |
| **B7** | **8.3994** | **7.8054** | **16.2048** | **8** | **0.5940** |
| **C1** | **8.3873** | **7.4798** | **15.8672** | **13** | **0.9075** |
| **C2** | **8.6703** | **7.8753** | **16.5457** | **6** | **0.7950** |
| **C3** | **7.6673** | **8.3624** | **16.0297** | **10** | **-0.6951** |
| **C4** | **7.7661** | **7.9972** | **15.7633** | **16** | **-0.2311** |
| **C5** | **8.3143** | **8.1760** | **16.4903** | **7** | **0.1382** |
| **C6** | **7.9562** | **8.2123** | **16.1684** | **9** | **-0.2561** |

This table lists the impact degree, influenced degree, centrality, and centrality ranking of all factors, ranking them by centrality.

**Step 7: Plotting the degree-of-causality-centrality graph**

The horizontal axis of a Degree-of-Causality-Centrality diagram is D+C and the vertical axis is D-C. The causality-centrality diagram can simplify the complex cause and effect relationship, which can help to analyze and solve the problem more intuitively and deeply. In addition, through this diagram, decision makers can clearly identify which factors belong to the "cause category" (i.e., factors that trigger changes in other factors) and which belong to the "effect category" (i.e., factors that are influenced by other factors). Based on these analyses, decision makers are able to make decisions and optimize them according to the characteristics of different types of factors. The result is shown in ***Fig S1***.


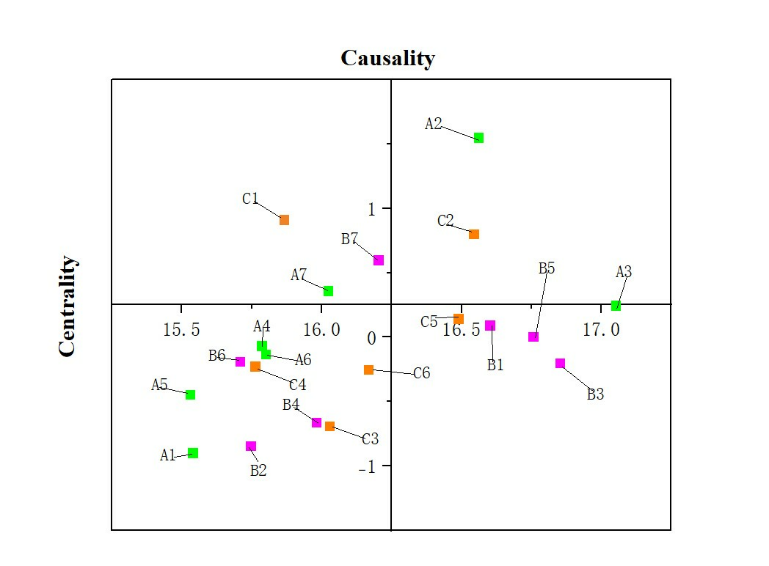


**Fig S1: Coal power supply chain elasticity cause-degree-centeredness diagram**

**B. Step-by-step execution of ISM**

**Step 1: Calculate the overall impact matrix**

The overall impact matrix was calculated according to Formula 6, and the results are shown in ***Table S7***.

**Table S7: The overall impact matrix**

|  | **A1** | **A2** | **A3** | **A4** | **A5** | **A6** | **A7** | **B1** | **B2** | **B3** | **B4** | **B5** | **B6** | **B7** | **C1** | **C2** | **C3** | **C4** | **C5** | **C6** |
| --- | --- | --- | --- | --- | --- | --- | --- | --- | --- | --- | --- | --- | --- | --- | --- | --- | --- | --- | --- | --- |
| **A1** | **1.3371** | **0.3439** | **0.3805** | **0.3698** | **0.3655** | **0.3718** | **0.3704** | **0.3698** | **0.3694** | **0.3927** | **0.3814** | **0.3968** | **0.3469** | **0.3519** | **0.3470** | **0.3611** | **0.3764** | **0.3501** | **0.3778** | **0.3584** |
| **A2** | **0.4607** | **1.3795** | **0.4739** | **0.4531** | **0.4407** | **0.4462** | **0.4399** | **0.4821** | **0.4471** | **0.4850** | **0.4687** | **0.4603** | **0.4520** | **0.4422** | **0.4207** | **0.4497** | **0.4744** | **0.4342** | **0.4604** | **0.4839** |
| **A3** | **0.4540** | **0.4034** | **1.4058** | **0.4390** | **0.4373** | **0.4419** | **0.4214** | **0.4307** | **0.4304** | **0.4769** | **0.4516** | **0.4627** | **0.4120** | **0.4287** | **0.4124** | **0.4147** | **0.4532** | **0.4260** | **0.4200** | **0.4257** |
| **A4** | **0.4034** | **0.3420** | **0.4115** | **1.3481** | **0.3879** | **0.3904** | **0.3847** | **0.4068** | **0.4103** | **0.4167** | **0.4017** | **0.3958** | **0.3895** | **0.3852** | **0.3630** | **0.4016** | **0.4207** | **0.3950** | **0.3976** | **0.4052** |
| **A5** | **0.3757** | **0.3618** | **0.4061** | **0.3709** | **1.3371** | **0.3765** | **0.3646** | **0.3910** | **0.3907** | **0.4002** | **0.3872** | **0.3809** | **0.3848** | **0.3612** | **0.3319** | **0.3728** | **0.3935** | **0.3716** | **0.3921** | **0.3895** |
| **A6** | **0.3961** | **0.3723** | **0.4174** | **0.3726** | **0.3773** | **1.3488** | **0.3771** | **0.4110** | **0.4150** | **0.4139** | **0.3895** | **0.4100** | **0.4039** | **0.3717** | **0.3602** | **0.3808** | **0.4046** | **0.3954** | **0.4008** | **0.4139** |
| **A7** | **0.4303** | **0.3822** | **0.4319** | **0.4126** | **0.4193** | **0.4077** | **1.3585** | **0.4095** | **0.4266** | **0.4375** | **0.4149** | **0.4283** | **0.3956** | **0.3981** | **0.3898** | **0.3989** | **0.4233** | **0.3976** | **0.4194** | **0.4071** |
| **B1** | **0.4194** | **0.3835** | **0.4196** | **0.4344** | **0.4187** | **0.4159** | **0.4013** | **1.3850** | **0.4434** | **0.4462** | **0.4413** | **0.4369** | **0.4180** | **0.3983** | **0.3867** | **0.3929** | **0.4239** | **0.4139** | **0.4352** | **0.4302** |
| **B2** | **0.3855** | **0.3532** | **0.3970** | **0.3801** | **0.3585** | **0.3507** | **0.3518** | **0.3827** | **1.3452** | **0.3747** | **0.3722** | **0.3849** | **0.3515** | **0.3621** | **0.3671** | **0.3667** | **0.3829** | **0.3879** | **0.3980** | **0.3960** |
| **B3** | **0.4318** | **0.3916** | **0.4314** | **0.4163** | **0.4050** | **0.4152** | **0.4031** | **0.4310** | **0.4397** | **1.3961** | **0.4472** | **0.4326** | **0.4123** | **0.4083** | **0.3732** | **0.3983** | **0.4295** | **0.4039** | **0.4321** | **0.4244** |
| **B4** | **0.3995** | **0.3579** | **0.4090** | **0.3824** | **0.3744** | **0.3755** | **0.3763** | **0.3897** | **0.3960** | **0.4062** | **1.3562** | **0.4041** | **0.3828** | **0.3750** | **0.3441** | **0.3847** | **0.3900** | **0.3892** | **0.3841** | **0.3810** |
| **B5** | **0.4409** | **0.3973** | **0.4403** | **0.3946** | **0.4147** | **0.4251** | **0.4057** | **0.4313** | **0.4289** | **0.4594** | **0.4266** | **1.3930** | **0.4197** | **0.4148** | **0.3908** | **0.4155** | **0.4427** | **0.3992** | **0.4218** | **0.4159** |
| **B6** | **0.3865** | **0.3646** | **0.4119** | **0.3755** | **0.3836** | **0.3821** | **0.3918** | **0.3963** | **0.3962** | **0.3978** | **0.4058** | **0.4154** | **1.3449** | **0.3774** | **0.3524** | **0.3891** | **0.4058** | **0.3832** | **0.3972** | **0.4008** |
| **B7** | **0.4351** | **0.3800** | **0.4196** | **0.4083** | **0.4202** | **0.4189** | **0.4239** | **0.4384** | **0.4261** | **0.4403** | **0.4422** | **0.4428** | **0.4163** | **1.3660** | **0.3979** | **0.4079** | **0.4478** | **0.4095** | **0.4289** | **0.4294** |
| **C1** | **0.4372** | **0.4012** | **0.4387** | **0.4213** | **0.4083** | **0.4189** | **0.4083** | **0.4300** | **0.4204** | **0.4427** | **0.4461** | **0.4333** | **0.4220** | **0.4110** | **1.3505** | **0.4057** | **0.4236** | **0.4236** | **0.4189** | **0.4254** |
| **C2** | **0.4320** | **0.4184** | **0.4480** | **0.4138** | **0.4450** | **0.4242** | **0.4221** | **0.4453** | **0.4491** | **0.4647** | **0.4483** | **0.4548** | **0.4350** | **0.4124** | **0.4094** | **1.3813** | **0.4433** | **0.4345** | **0.4478** | **0.4409** |
| **C3** | **0.3817** | **0.3564** | **0.4036** | **0.3775** | **0.3944** | **0.3775** | **0.3671** | **0.3770** | **0.4053** | **0.3929** | **0.4054** | **0.4080** | **0.3834** | **0.3663** | **0.3506** | **0.3788** | **1.3585** | **0.4060** | **0.3884** | **0.3886** |
| **C4** | **0.3909** | **0.3632** | **0.4034** | **0.3746** | **0.3961** | **0.3864** | **0.3627** | **0.4102** | **0.4166** | **0.4189** | **0.3974** | **0.3951** | **0.3749** | **0.3683** | **0.3742** | **0.3800** | **0.4073** | **1.3478** | **0.3826** | **0.4155** |
| **C5** | **0.4116** | **0.3786** | **0.4328** | **0.4005** | **0.4077** | **0.3996** | **0.4064** | **0.4282** | **0.4355** | **0.4494** | **0.4410** | **0.4322** | **0.4141** | **0.4143** | **0.3907** | **0.4018** | **0.4443** | **0.4304** | **1.3796** | **0.4154** |
| **C6** | **0.4130** | **0.3773** | **0.4229** | **0.3849** | **0.3989** | **0.3955** | **0.3981** | **0.4120** | **0.4074** | **0.4178** | **0.3997** | **0.4114** | **0.3918** | **0.3921** | **0.3673** | **0.3929** | **0.4168** | **0.3981** | **0.3933** | **1.3651** |

**Step 2: Calculate the reachable matrix** $\boldsymbol{K}$**.**

To determine the threshold value λ, it is calculated based on a statistical distribution method as: $\lambda=\mu+\nu$. Here, μ and ν represent the mean and standard deviation of all elements in matrix T.

The value of λ is calculated as 0.4331 using Equations 7 and 8.

The reachable matrix K is then obtained using Equation 9. The results are shown in ***Table S8***.

**Table S8: Coal Power Supply Chain Resilience Influencing Factors Reachable Matrix K**

|  | **A1** | **A2** | **A3** | **A4** | **A5** | **A6** | **A7** | **B1** | **B2** | **B3** | **B4** | **B5** | **B6** | **B7** | **C1** | **C2** | **C3** | **C4** | **C5** | **C6** |
| --- | --- | --- | --- | --- | --- | --- | --- | --- | --- | --- | --- | --- | --- | --- | --- | --- | --- | --- | --- | --- |
| **A1** | **1** | **0** | **0** | **0** | **0** | **0** | **0** | **0** | **0** | **0** | **0** | **0** | **0** | **0** | **0** | **0** | **0** | **0** | **0** | **0** |
| **A2** | **1** | **1** | **1** | **1** | **1** | **1** | **1** | **1** | **1** | **1** | **1** | **1** | **1** | **1** | **0** | **1** | **1** | **1** | **1** | **1** |
| **A3** | **1** | **0** | **1** | **1** | **1** | **1** | **0** | **0** | **1** | **1** | **1** | **1** | **0** | **0** | **0** | **0** | **1** | **0** | **0** | **0** |
| **A4** | **0** | **0** | **0** | **1** | **0** | **0** | **0** | **0** | **0** | **0** | **0** | **0** | **0** | **0** | **0** | **0** | **0** | **0** | **0** | **0** |
| **A5** | **0** | **0** | **0** | **0** | **1** | **0** | **0** | **0** | **0** | **0** | **0** | **0** | **0** | **0** | **0** | **0** | **0** | **0** | **0** | **0** |
| **A6** | **0** | **0** | **0** | **0** | **0** | **1** | **0** | **0** | **0** | **0** | **0** | **0** | **0** | **0** | **0** | **0** | **0** | **0** | **0** | **0** |
| **A7** | **0** | **0** | **0** | **0** | **0** | **0** | **1** | **0** | **1** | **1** | **1** | **0** | **0** | **0** | **0** | **0** | **0** | **0** | **0** | **0** |
| **B1** | **1** | **0** | **1** | **1** | **1** | **1** | **0** | **1** | **1** | **1** | **1** | **1** | **0** | **0** | **0** | **0** | **1** | **0** | **1** | **0** |
| **B2** | **0** | **0** | **0** | **0** | **0** | **0** | **0** | **0** | **1** | **0** | **0** | **0** | **0** | **0** | **0** | **0** | **0** | **0** | **0** | **0** |
| **B3** | **0** | **0** | **0** | **0** | **0** | **0** | **0** | **0** | **1** | **1** | **1** | **0** | **0** | **0** | **0** | **0** | **0** | **0** | **0** | **0** |
| **B4** | **0** | **0** | **0** | **0** | **0** | **0** | **0** | **0** | **0** | **0** | **1** | **0** | **0** | **0** | **0** | **0** | **0** | **0** | **0** | **0** |
| **B5** | **1** | **0** | **1** | **1** | **1** | **1** | **0** | **0** | **1** | **1** | **1** | **1** | **0** | **0** | **0** | **0** | **1** | **0** | **0** | **0** |
| **B6** | **0** | **0** | **0** | **0** | **0** | **0** | **0** | **0** | **0** | **0** | **0** | **0** | **1** | **0** | **0** | **0** | **0** | **0** | **0** | **0** |
| **B7** | **1** | **0** | **1** | **1** | **1** | **1** | **0** | **1** | **1** | **1** | **1** | **1** | **0** | **1** | **0** | **0** | **1** | **0** | **1** | **0** |
| **C1** | **1** | **0** | **1** | **1** | **1** | **1** | **0** | **0** | **1** | **1** | **1** | **1** | **0** | **0** | **1** | **0** | **1** | **0** | **0** | **0** |
| **C2** | **1** | **0** | **1** | **1** | **1** | **1** | **0** | **1** | **1** | **1** | **1** | **1** | **1** | **0** | **0** | **1** | **1** | **1** | **1** | **1** |
| **C3** | **0** | **0** | **0** | **0** | **0** | **0** | **0** | **0** | **0** | **0** | **0** | **0** | **0** | **0** | **0** | **0** | **1** | **0** | **0** | **0** |
| **C4** | **0** | **0** | **0** | **0** | **0** | **0** | **0** | **0** | **0** | **0** | **0** | **0** | **0** | **0** | **0** | **0** | **0** | **1** | **0** | **0** |
| **C5** | **0** | **0** | **0** | **0** | **0** | **0** | **0** | **0** | **1** | **1** | **1** | **0** | **0** | **0** | **0** | **0** | **1** | **0** | **1** | **0** |
| **C6** | **0** | **0** | **0** | **0** | **0** | **0** | **0** | **0** | **0** | **0** | **0** | **0** | **0** | **0** | **0** | **0** | **0** | **0** | **0** | **1** |

**Step 3: Determine the reachable set Q, the prior set A, and the intersection set to construct a multilevel recursive order structure model of the influencing factors.**

Calculate the reachability set Q, the prior set A and the intersection set according to Formulas 10 and 11. The calculation results are shown in ***Table S9***.

**Table S9: Reachable Set, Antecedent Set, and Intersection Set for the First Level**

| Indicators | Reachable set | Precedence set | Intersection |
| --- | --- | --- | --- |
| A1 | [A1] | [A1, A2, A3, B1, B5, B7, C1, C2] | [A1] |
| A2 | [A1, A2, A3, A4, A5, A6, A7, B1, B2, B3, B4, B5, B6, B7, C2, C3, C4, C5, C6] | [A2] | [A2] |
| A3 | [A1, A3, A4, A5, A6, B2, B3, B4, B5, C3] | [A2, A3, B1, B5, B7, C1, C2] | [A3, B5] |
| A4 | [A4] | [A2, A3, A4, B1, B5, B7, C1, C2] | [A4] |
| A5 | [A5] | [A2, A3, A5, B1, B5, B7, C1, C2] | [A5] |
| A6 | [A6] | [A2, A3, A6, B1, B5, B7, C1, C2] | [A6] |
| A7 | [A7, B2, B3, B4] | [A2, A7] | [A7] |
| B1 | [A1, A3, A4, A5, A6, B1, B2, B3, B4, B5, C3, C5] | [A2, B1, B7, C2] | [B1] |
| B2 | [B2] | [A2, A3, A7, B1, B3, B5, B7, C1, C2, C5] | [B2] |
| B3 | [B2, B3, B4] | [A2, A3, A7, B1, B3, B5, B7, C1, C2, C5, C6] | [B3] |
| B4 | [B4] | [A2, A3, A7, B1, B3, B4, B5, B7, C1, C2, C5] | [B4] |
| B5 | [A1, A3, A4, A5, A6, B2, B3, B4, B5, C3] | [A2, A3, B1, B5, B7, C1, C2] | [A3, B5] |
| B6 | [B6] | [A2, B6, C2] | [B6] |
| B7 | [A1, A3, A4, A5, A6, B1, B2, B3, B4, B5, B7, C3, C5] | [A2, B7] | [B7] |
| C1 | [A1, A3, A4, A5, A6, B2, B3, B4, B5, C1, C3] | [C1] | [C1] |
| C2 | [A1, A3, A4, A5, A6, B1, B2, B3, B4, B5, B6, C2, C3, C4, C5, C6] | [A2, C2] | [C2] |
| C3 | [C3] | [A2, A3, B1, B5, B7, C1, C2, C3, C5] | [C3] |
| C4 | [C4] | [A2, C2, C4] | [C4] |
| C5 | [B2, B3, B4, C3, C5] | [A2, B1, B7, C2, C5] | [C5] |
| C6 | [C6] | [A2, C2, C6] | [C6] |

From the above table, it can be determined that the first-level influencing factors include A1, A4, A5, A6, B2, B4, B6, C3, C4, and C6. The corresponding rows and columns for these factors should be removed from the table. Continue the calculation. The calculation results are shown in ***Table S10***.

**Table S10: Reachable Set, Antecedent Set, and Intersection Set for the Second Level**

| Indicators | Reachable set | Precedence set | Intersection |
| --- | --- | --- | --- |
| A2 | [ A2, A3, A7, B1, B3, B5, B7, C2, C5] | [A2] | [A2] |
| A3 | [A3, B3, B5] | [A2, A3, B1, B5, B7, C1, C2] | [A3, B5] |
| A7 | [A7, B3] | [A2, A7] | [A7] |
| B1 | [A3, B1, B3, B5, C5] | [A2, B1, B7, C2] | [B1] |
| B3 | [B3] | [A2, A3, A7, B1, B3, B5, B7, C1, C2, C5] | [B3] |
| B5 | [ A3, B3, B5] | [A2, A3, B1, B5, B7, C1, C2] | [A3, B5] |
| B7 | [A3, B1, B3, B5, B7, C5] | [A2, B7] | [B7] |
| C1 | [A3, B3, B5, C1] | [C1] | [C1] |
| C2 | [A3, B1, B3, B5, C2, C5] | [A2, C2] | [C2] |
| C5 | [B3, C5] | [A2, B1, B7, C2, C5] | [C5] |

The second-level influencing factor includes B3. The corresponding row and column for the B3 influencing factor should be removed from the table. Continue the calculation. The calculation results are shown in ***Table S11***.

**Table S11: Reachable Set, Antecedent Set, and Intersection Set for the Third Level**

| Indicators | Reachable set | Precedence set | Intersection |
| --- | --- | --- | --- |
| A2 | [ A2, A3, A7, B1, B5, B7, C2, C5] | [A2] | [A2] |
| A3 | [A3, B5] | [A2, A3, B1, B5, B7, C1, C2] | [A3, B5] |
| A7 | [A7] | [A2, A7] | [A7] |
| B1 | [A3, B1, B5, C5] | [A2, B1, B7, C2] | [B1] |
| B5 | [ A3, B5] | [A2, A3, B1, B5, B7, C1, C2] | [A3, B5] |
| B7 | [A3, B1, B5, B7, C5] | [A2, B7] | [B7] |
| C1 | [A3, B5, C1] | [C1] | [C1] |
| C2 | [A3, B1, B5, C2, C5] | [A2, C2] | [C2] |
| C5 | [C5] | [A2, B1, B7, C2, C5] | [C5] |

The third-level influencing factors include A3, A7, B5, and C5. The corresponding rows and columns for these four influencing factors should be removed from the table, and the layering process should continue. The calculation results are shown in ***Table S12***.

**Table S12: Reachable Set, Antecedent Set, and Intersection Set for the Fourth Level**

| Indicators | Reachable set | Precedence set | Intersection |
| --- | --- | --- | --- |
| A2 | [ A2, B1, B7, C2] | [A2] | [A2] |
| B1 | [B1] | [A2, B1, B7, C2] | [B1] |
| B7 | [B1, B7] | [A2, B7] | [B7] |
| C1 | [C1] | [C1] | [C1] |
| C2 | [B1, C2] | [A2, C2] | [C2] |

The fourth-level influencing factors include B1 and C1. The corresponding rows and columns for these two influencing factors should be removed from the table, and the layering process should continue. The calculation results are shown in ***Table S13***.

**Table S13：Reachable Set, Antecedent Set, and Intersection Set for the Fifth Level**

| Indicators | Reachable set | Precedence set | Intersection |
| --- | --- | --- | --- |
| A2 | [ A2, B7, C2] | [A2] | [A2] |
| B7 | [B7] | [A2, B7] | [B7] |
| C2 | [C2] | [A2, C2] | [C2] |

The fifth-level influencing factors include B7 and C2. The corresponding rows and columns for these two influencing factors should be removed from the table, and the layering process should continue. The calculation results are shown in ***Table S14***.

**Table S14: Reachable Set, Antecedent Set, and Intersection Set for the Sixth Level**

| Indicators | Reachable set | Precedence set | Intersection |
| --- | --- | --- | --- |
| A2 | [A2] | [A2] | [A2] |

The sixth-level influencing factor is A2, marking the conclusion of the UP hierarchical structuring process. The hierarchical levels are as follows: First Level (L1): {A1, A4, A5, A6, B2, B4, B6, C3, C4, C6}; Second Level (L2): {B3}; Third Level (L3): {A3, A7, B5, C5}; Fourth Level (L4): {B1, C1}; Fifth Level (L5): {B7, C2}; Sixth Level (L6): {A2}.

**Step 4: Constructing the hierarchical structure diagram**

Construct the hierarchical structure diagram based on the hierarchical structure obtained in Step 3. The constructed hierarchical structure diagram is shown in ***Fig S2***.


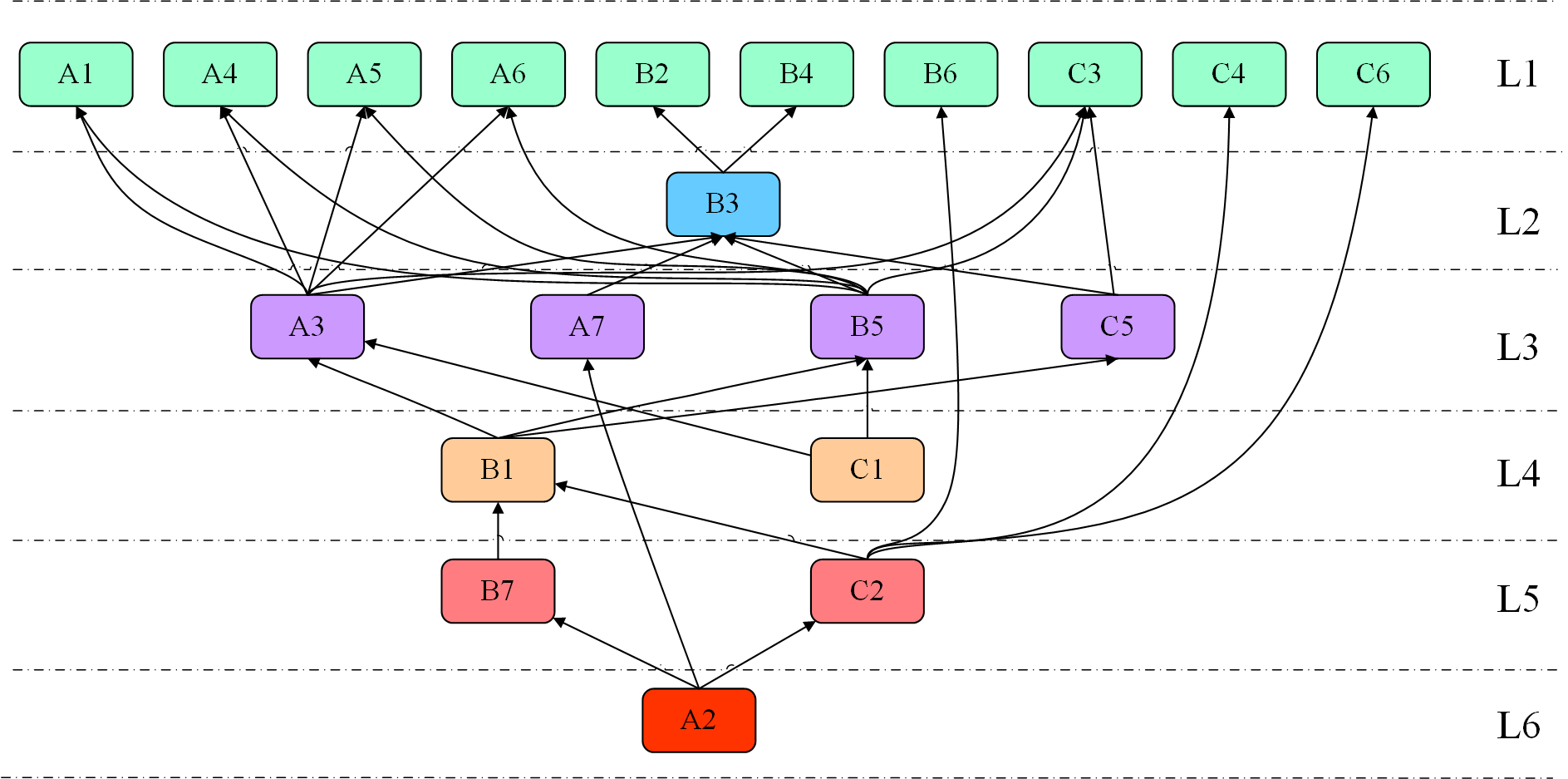


**Fig S2Hierarchical Structure Diagram of Influencing Factors in Coal Power Supply Chain Resilience**

C. Step-by-step execution of ANP

**Step 1: Calculate the weighted supermatrix.**

The overall impact matrix T is considered as an unweighted supermatrix, which is then normalized to obtain the weighted supermatrix W. The calculation results are shown in ***Table S15***.

**Table S15: The unweighted supermatrix**

|  | **A1** | **A2** | **A3** | **A4** | **A5** | **A6** | **A7** | **B1** | **B2** | **B3** | **B4** | **B5** | **B6** | **B7** | **C1** | **C2** | **C3** | **C4** | **C5** | **C6** |
| --- | --- | --- | --- | --- | --- | --- | --- | --- | --- | --- | --- | --- | --- | --- | --- | --- | --- | --- | --- | --- |
| **A1** | **0.0410** | **0.0458** | **0.0453** | **0.0466** | **0.0457** | **0.0467** | **0.0473** | **0.0448** | **0.0445** | **0.0460** | **0.0458** | **0.0474** | **0.0436** | **0.0451** | **0.0464** | **0.0459** | **0.0450** | **0.0438** | **0.0462** | **0.0436** |
| **A2** | **0.0560** | **0.0505** | **0.0564** | **0.0571** | **0.0552** | **0.0560** | **0.0561** | **0.0584** | **0.0539** | **0.0569** | **0.0563** | **0.0549** | **0.0568** | **0.0567** | **0.0562** | **0.0571** | **0.0567** | **0.0543** | **0.0563** | **0.0589** |
| **A3** | **0.0552** | **0.0537** | **0.0483** | **0.0554** | **0.0547** | **0.0555** | **0.0538** | **0.0522** | **0.0519** | **0.0559** | **0.0543** | **0.0552** | **0.0518** | **0.0549** | **0.0551** | **0.0527** | **0.0542** | **0.0533** | **0.0514** | **0.0518** |
| **A4** | **0.0491** | **0.0455** | **0.0490** | **0.0439** | **0.0485** | **0.0490** | **0.0491** | **0.0493** | **0.0494** | **0.0489** | **0.0483** | **0.0472** | **0.0490** | **0.0494** | **0.0485** | **0.0510** | **0.0503** | **0.0494** | **0.0486** | **0.0493** |
| **A5** | **0.0457** | **0.0482** | **0.0483** | **0.0468** | **0.0422** | **0.0472** | **0.0465** | **0.0473** | **0.0471** | **0.0469** | **0.0465** | **0.0455** | **0.0484** | **0.0463** | **0.0444** | **0.0473** | **0.0471** | **0.0465** | **0.0480** | **0.0474** |
| **A6** | **0.0482** | **0.0496** | **0.0497** | **0.0470** | **0.0472** | **0.0438** | **0.0481** | **0.0498** | **0.0500** | **0.0485** | **0.0468** | **0.0489** | **0.0508** | **0.0476** | **0.0482** | **0.0484** | **0.0484** | **0.0494** | **0.0490** | **0.0504** |
| **A7** | **0.0523** | **0.0509** | **0.0514** | **0.0520** | **0.0525** | **0.0512** | **0.0458** | **0.0496** | **0.0514** | **0.0513** | **0.0498** | **0.0511** | **0.0498** | **0.0510** | **0.0521** | **0.0507** | **0.0506** | **0.0497** | **0.0513** | **0.0496** |
| **B1** | **0.0510** | **0.0511** | **0.0499** | **0.0548** | **0.0524** | **0.0522** | **0.0512** | **0.0466** | **0.0534** | **0.0523** | **0.0530** | **0.0521** | **0.0526** | **0.0510** | **0.0517** | **0.0499** | **0.0507** | **0.0518** | **0.0532** | **0.0524** |
| **B2** | **0.0469** | **0.0470** | **0.0472** | **0.0479** | **0.0449** | **0.0440** | **0.0449** | **0.0463** | **0.0416** | **0.0439** | **0.0447** | **0.0459** | **0.0442** | **0.0464** | **0.0491** | **0.0466** | **0.0458** | **0.0485** | **0.0487** | **0.0482** |
| **B3** | **0.0525** | **0.0522** | **0.0513** | **0.0525** | **0.0507** | **0.0521** | **0.0514** | **0.0522** | **0.0530** | **0.0464** | **0.0537** | **0.0516** | **0.0519** | **0.0523** | **0.0499** | **0.0506** | **0.0514** | **0.0505** | **0.0528** | **0.0517** |
| **B4** | **0.0486** | **0.0477** | **0.0487** | **0.0482** | **0.0469** | **0.0471** | **0.0480** | **0.0472** | **0.0477** | **0.0476** | **0.0428** | **0.0482** | **0.0481** | **0.0480** | **0.0460** | **0.0488** | **0.0466** | **0.0487** | **0.0470** | **0.0464** |
| **B5** | **0.0536** | **0.0529** | **0.0524** | **0.0498** | **0.0519** | **0.0533** | **0.0518** | **0.0522** | **0.0517** | **0.0539** | **0.0512** | **0.0469** | **0.0528** | **0.0531** | **0.0522** | **0.0528** | **0.0529** | **0.0499** | **0.0516** | **0.0506** |
| **B6** | **0.0470** | **0.0486** | **0.0490** | **0.0474** | **0.0480** | **0.0479** | **0.0500** | **0.0480** | **0.0477** | **0.0466** | **0.0487** | **0.0496** | **0.0434** | **0.0484** | **0.0471** | **0.0494** | **0.0485** | **0.0479** | **0.0486** | **0.0488** |
| **B7** | **0.0529** | **0.0506** | **0.0499** | **0.0515** | **0.0526** | **0.0526** | **0.0541** | **0.0531** | **0.0513** | **0.0516** | **0.0531** | **0.0528** | **0.0524** | **0.0469** | **0.0532** | **0.0518** | **0.0535** | **0.0512** | **0.0525** | **0.0523** |
| **C1** | **0.0532** | **0.0534** | **0.0522** | **0.0531** | **0.0511** | **0.0526** | **0.0521** | **0.0521** | **0.0507** | **0.0519** | **0.0536** | **0.0517** | **0.0531** | **0.0527** | **0.0469** | **0.0515** | **0.0507** | **0.0530** | **0.0512** | **0.0518** |
| **C2** | **0.0525** | **0.0557** | **0.0533** | **0.0522** | **0.0557** | **0.0532** | **0.0539** | **0.0539** | **0.0541** | **0.0545** | **0.0539** | **0.0543** | **0.0547** | **0.0528** | **0.0547** | **0.0484** | **0.0530** | **0.0543** | **0.0548** | **0.0537** |
| **C3** | **0.0464** | **0.0475** | **0.0480** | **0.0476** | **0.0494** | **0.0474** | **0.0469** | **0.0457** | **0.0488** | **0.0461** | **0.0487** | **0.0487** | **0.0482** | **0.0469** | **0.0469** | **0.0481** | **0.0429** | **0.0508** | **0.0475** | **0.0473** |
| **C4** | **0.0475** | **0.0484** | **0.0480** | **0.0472** | **0.0496** | **0.0485** | **0.0463** | **0.0497** | **0.0502** | **0.0491** | **0.0477** | **0.0472** | **0.0471** | **0.0472** | **0.0500** | **0.0483** | **0.0487** | **0.0435** | **0.0468** | **0.0506** |
| **C5** | **0.0501** | **0.0504** | **0.0515** | **0.0505** | **0.0510** | **0.0501** | **0.0519** | **0.0519** | **0.0525** | **0.0527** | **0.0530** | **0.0516** | **0.0521** | **0.0531** | **0.0522** | **0.0510** | **0.0531** | **0.0538** | **0.0464** | **0.0506** |
| **C6** | **0.0502** | **0.0503** | **0.0503** | **0.0485** | **0.0499** | **0.0496** | **0.0508** | **0.0499** | **0.0491** | **0.0490** | **0.0480** | **0.0491** | **0.0493** | **0.0502** | **0.0491** | **0.0499** | **0.0498** | **0.0498** | **0.0481** | **0.0445** |

**Step 2: Calculate the limiting supermatrix**

The resulting weighted supermatrix is then used to calculate the limiting supermatrix by using formula 13. The calculation results are shown in ***Table S16***.

**Table S16: The limiting supermatrix**

|  | **A1** | **A2** | **A3** | **A4** | **A5** | **A6** | **A7** | **B1** | **B2** | **B3** | **B4** | **B5** | **B6** | **B7** | **C1** | **C2** | **C3** | **C4** | **C5** | **C6** |
| --- | --- | --- | --- | --- | --- | --- | --- | --- | --- | --- | --- | --- | --- | --- | --- | --- | --- | --- | --- | --- |
| **A1** | **0.0454** | **0.0454** | **0.0454** | **0.0454** | **0.0454** | **0.0454** | **0.0454** | **0.0454** | **0.0454** | **0.0454** | **0.0454** | **0.0454** | **0.0454** | **0.0454** | **0.0454** | **0.0454** | **0.0454** | **0.0454** | **0.0454** | **0.0454** |
| **A2** | **0.0560** | **0.0560** | **0.0560** | **0.0560** | **0.0560** | **0.0560** | **0.0560** | **0.0560** | **0.0560** | **0.0560** | **0.0560** | **0.0560** | **0.0560** | **0.0560** | **0.0560** | **0.0560** | **0.0560** | **0.0560** | **0.0560** | **0.0560** |
| **A3** | **0.0535** | **0.0535** | **0.0535** | **0.0535** | **0.0535** | **0.0535** | **0.0535** | **0.0535** | **0.0535** | **0.0535** | **0.0535** | **0.0535** | **0.0535** | **0.0535** | **0.0535** | **0.0535** | **0.0535** | **0.0535** | **0.0535** | **0.0535** |
| **A4** | **0.0486** | **0.0486** | **0.0486** | **0.0486** | **0.0486** | **0.0486** | **0.0486** | **0.0486** | **0.0486** | **0.0486** | **0.0486** | **0.0486** | **0.0486** | **0.0486** | **0.0486** | **0.0486** | **0.0486** | **0.0486** | **0.0486** | **0.0486** |
| **A5** | **0.0467** | **0.0467** | **0.0467** | **0.0467** | **0.0467** | **0.0467** | **0.0467** | **0.0467** | **0.0467** | **0.0467** | **0.0467** | **0.0467** | **0.0467** | **0.0467** | **0.0467** | **0.0467** | **0.0467** | **0.0467** | **0.0467** | **0.0467** |
| **A6** | **0.0485** | **0.0485** | **0.0485** | **0.0485** | **0.0485** | **0.0485** | **0.0485** | **0.0485** | **0.0485** | **0.0485** | **0.0485** | **0.0485** | **0.0485** | **0.0485** | **0.0485** | **0.0485** | **0.0485** | **0.0485** | **0.0485** | **0.0485** |
| **A7** | **0.0507** | **0.0507** | **0.0507** | **0.0507** | **0.0507** | **0.0507** | **0.0507** | **0.0507** | **0.0507** | **0.0507** | **0.0507** | **0.0507** | **0.0507** | **0.0507** | **0.0507** | **0.0507** | **0.0507** | **0.0507** | **0.0507** | **0.0507** |
| **B1** | **0.0516** | **0.0516** | **0.0516** | **0.0516** | **0.0516** | **0.0516** | **0.0516** | **0.0516** | **0.0516** | **0.0516** | **0.0516** | **0.0516** | **0.0516** | **0.0516** | **0.0516** | **0.0516** | **0.0516** | **0.0516** | **0.0516** | **0.0516** |
| **B2** | **0.0462** | **0.0462** | **0.0462** | **0.0462** | **0.0462** | **0.0462** | **0.0462** | **0.0462** | **0.0462** | **0.0462** | **0.0462** | **0.0462** | **0.0462** | **0.0462** | **0.0462** | **0.0462** | **0.0462** | **0.0462** | **0.0462** | **0.0462** |
| **B3** | **0.0515** | **0.0515** | **0.0515** | **0.0515** | **0.0515** | **0.0515** | **0.0515** | **0.0515** | **0.0515** | **0.0515** | **0.0515** | **0.0515** | **0.0515** | **0.0515** | **0.0515** | **0.0515** | **0.0515** | **0.0515** | **0.0515** | **0.0515** |
| **B4** | **0.0474** | **0.0474** | **0.0474** | **0.0474** | **0.0474** | **0.0474** | **0.0474** | **0.0474** | **0.0474** | **0.0474** | **0.0474** | **0.0474** | **0.0474** | **0.0474** | **0.0474** | **0.0474** | **0.0474** | **0.0474** | **0.0474** | **0.0474** |
| **B5** | **0.0519** | **0.0519** | **0.0519** | **0.0519** | **0.0519** | **0.0519** | **0.0519** | **0.0519** | **0.0519** | **0.0519** | **0.0519** | **0.0519** | **0.0519** | **0.0519** | **0.0519** | **0.0519** | **0.0519** | **0.0519** | **0.0519** | **0.0519** |
| **B6** | **0.0481** | **0.0481** | **0.0481** | **0.0481** | **0.0481** | **0.0481** | **0.0481** | **0.0481** | **0.0481** | **0.0481** | **0.0481** | **0.0481** | **0.0481** | **0.0481** | **0.0481** | **0.0481** | **0.0481** | **0.0481** | **0.0481** | **0.0481** |
| **B7** | **0.0520** | **0.0520** | **0.0520** | **0.0520** | **0.0520** | **0.0520** | **0.0520** | **0.0520** | **0.0520** | **0.0520** | **0.0520** | **0.0520** | **0.0520** | **0.0520** | **0.0520** | **0.0520** | **0.0520** | **0.0520** | **0.0520** | **0.0520** |
| **C1** | **0.0519** | **0.0519** | **0.0519** | **0.0519** | **0.0519** | **0.0519** | **0.0519** | **0.0519** | **0.0519** | **0.0519** | **0.0519** | **0.0519** | **0.0519** | **0.0519** | **0.0519** | **0.0519** | **0.0519** | **0.0519** | **0.0519** | **0.0519** |
| **C2** | **0.0537** | **0.0537** | **0.0537** | **0.0537** | **0.0537** | **0.0537** | **0.0537** | **0.0537** | **0.0537** | **0.0537** | **0.0537** | **0.0537** | **0.0537** | **0.0537** | **0.0537** | **0.0537** | **0.0537** | **0.0537** | **0.0537** | **0.0537** |
| **C3** | **0.0475** | **0.0475** | **0.0475** | **0.0475** | **0.0475** | **0.0475** | **0.0475** | **0.0475** | **0.0475** | **0.0475** | **0.0475** | **0.0475** | **0.0475** | **0.0475** | **0.0475** | **0.0475** | **0.0475** | **0.0475** | **0.0475** | **0.0475** |
| **C4** | **0.0481** | **0.0481** | **0.0481** | **0.0481** | **0.0481** | **0.0481** | **0.0481** | **0.0481** | **0.0481** | **0.0481** | **0.0481** | **0.0481** | **0.0481** | **0.0481** | **0.0481** | **0.0481** | **0.0481** | **0.0481** | **0.0481** | **0.0481** |
| **C5** | **0.0515** | **0.0515** | **0.0515** | **0.0515** | **0.0515** | **0.0515** | **0.0515** | **0.0515** | **0.0515** | **0.0515** | **0.0515** | **0.0515** | **0.0515** | **0.0515** | **0.0515** | **0.0515** | **0.0515** | **0.0515** | **0.0515** | **0.0515** |
| **C6** | **0.0493** | **0.0493** | **0.0493** | **0.0493** | **0.0493** | **0.0493** | **0.0493** | **0.0493** | **0.0493** | **0.0493** | **0.0493** | **0.0493** | **0.0493** | **0.0493** | **0.0493** | **0.0493** | **0.0493** | **0.0493** | **0.0493** | **0.0493** |

According to the limiting supermatrix obtained, the weights of each influential factor of coal power supply chain elasticity can be obtained. The results are shown in ***Table S17***.

**Table S17: Weights of factors influencing the resilience of the coal power supply chain**

| Primary Indicators | Primary Indicators weight | Secondary Indicators | Secondary Indicators weight | Weight sorting |
| --- | --- | --- | --- | --- |
| Restorative Capacity | 0.3494 | A1 | 0.0454 | 20 |
|  |  | A2 | 0.0560 | 1 |
|  |  | A3 | 0.0535 | 3 |
|  |  | A4 | 0.0486 | 12 |
|  |  | A5 | 0.0467 | 18 |
|  |  | A6 | 0.0485 | 13 |
|  |  | A7 | 0.0507 | 10 |
| Absorptive Capacity | 0.3487 | B1 | 0.0516 | 7 |
|  |  | B2 | 0.0462 | 19 |
|  |  | B3 | 0.0515 | 8 |
|  |  | B4 | 0.0474 | 17 |
|  |  | B5 | 0.0519 | 6 |
|  |  | B6 | 0.0481 | 15 |
|  |  | B7 | 0.0520 | 4 |
| Adaptive Capacity | 0.3019 | C1 | 0.0519 | 5 |
|  |  | C2 | 0.0537 | 2 |
|  |  | C3 | 0.0475 | 16 |
|  |  | C4 | 0.0481 | 14 |
|  |  | C5 | 0.0515 | 9 |
|  |  | C6 | 0.0493 | 11 |
